# Xyloglucan Remodeling Defines Auxin-Dependent Differential Tissue Expansion in Plants

**DOI:** 10.3390/ijms22179222

**Published:** 2021-08-26

**Authors:** Silvia Melina Velasquez, Xiaoyuan Guo, Marçal Gallemi, Bibek Aryal, Peter Venhuizen, Elke Barbez, Kai Alexander Dünser, Martin Darino, Aleš Pĕnčík, Ondřej Novák, Maria Kalyna, Gregory Mouille, Eva Benková, Rishikesh P. Bhalerao, Jozef Mravec, Jürgen Kleine-Vehn

**Affiliations:** 1 Department of Applied Genetics and Cell Biology, University of Natural Resources and Life Sciences Vienna (BOKU), Muthgasse 18, 1190 Vienna, Austria; peter.venhuizen@boku.ac.at (P.V.); elkebarbez@gmail.com (E.B.); kai.duenser@boku.ac.at (K.A.D.); martin.a.darino@gmail.com (M.D.); mariya.kalyna@boku.ac.at (M.K.); 2 Department of Plant and Environmental Sciences, University of Copenhagen, Thorvaldsensvej 40, DK-1871 Frederiksberg C, Denmark; yuanquane@gmail.com (X.G.); mravec@plen.ku.dk (J.M.); 3 Institute of Science and Technology Austria, 3400 Klosterneuburg, Austria; marcal.gallemi@ist.ac.at (M.G.); eva.benkova@ist.ac.at (E.B.); 4 Department of Forest Genetics and Plant Physiology, Umeå Plant Science Centre, Swedish University of Agricultural Sciences, SE-901 87 Umeå, Sweden; Bibek.aryal@slu.se (B.A.); ondrej.novak@upol.cz (O.N.); Rishi.Bhalerao@slu.se (R.P.B.); 5 Faculty of Biology, Department of Molecular Plant Physiology (MoPP), University of Freiburg, 79104 Freiburg, Germany; 6 Laboratory of Growth Regulators, Faculty of Science, Palacký University and Institute of Experimental Botany, The Czech Academy of Sciences, Šlechtitelů 27, 78371 Olomouc, Czech Republic; alespencik@seznam.cz; 7 Institut Jean-Pierre Bourgin, Institut National de la Recherche Agronomique, AgroParisTech, CNRS, Université Paris-Saclay, RD10, CEDEX, 78026 Versailles, France; gregory.mouille@inra.fr; 8 Center for Integrative Biological Signalling Studies (CIBSS), University of Freiburg, 79104 Freiburg, Germany

**Keywords:** auxin, growth, cell wall, xyloglucans, hypocotyls, gravitropism

## Abstract

Size control is a fundamental question in biology, showing incremental complexity in plants, whose cells possess a rigid cell wall. The phytohormone auxin is a vital growth regulator with central importance for differential growth control. Our results indicate that auxin-reliant growth programs affect the molecular complexity of xyloglucans, the major type of cell wall hemicellulose in eudicots. Auxin-dependent induction and repression of growth coincide with reduced and enhanced molecular complexity of xyloglucans, respectively. In agreement with a proposed function in growth control, genetic interference with xyloglucan side decorations distinctly modulates auxin-dependent differential growth rates. Our work proposes that auxin-dependent growth programs have a spatially defined effect on xyloglucan’s molecular structure, which in turn affects cell wall mechanics and specifies differential, gravitropic hypocotyl growth.

## 1. Introduction

The phytohormone auxin is a central regulator of plant development and is of pivotal importance for differential growth control. Despite its significance, we do not fully understand the subcellular mechanisms by which auxin reliant growth programs define the size of a cell that is surrounded by a rigid cell wall structure. Auxin signaling steers promotion and repression of cell expansion in a concentration- and cell-type-dependent manner [1]. The cellular levels of auxin rely on a complex interplay between metabolism and intercellular transport [2,3,4]. On the other hand, tissue specific expression of auxin signaling components and intracellular auxin transport define cellular sensitivity to auxins [5,6,7]. Transcriptional auxin responses take place in the nucleus via auxin binding to its co-receptors transport inhibitor response 1/auxin signaling F-box (TIR1/AFBs) and the transcriptional repressor auxin/indole-3-acetic acid (Aux/IAAs) [8]. Auxin-induced cellular elongation in hypocotyls requires TIR1/AFBs-dependent transcriptional auxin responses [9]. In contrast, auxin-triggered repression of root cell expansion utilizes a TIR1/AFBs-dependent, non-genomic pathway [10]. In addition, the receptor like kinase TRANSMEMBRANE RECEPETOR LIKE KINASE1 (TMK1) is required for a non-genomic effect of auxin in apical hooks, repressing growth at the inner side (concave) [11]. On the other hand, TMK1 does not impact on hypocotyl expansion [12]. These findings suggest a complex and tissue specific interplay of auxin signaling and growth. 

Auxin-dependent control of cellular expansion is in part manifested by stiffening or loosening of the primary cell wall [13], but the underlying molecular mechanisms remain long-standing research questions. The plant cell wall in eudicots is a complex composite structure comprised mainly of polysaccharides, such as cellulose microfibrils, branched xyloglucans (XyG), arabinoxylans, mannans, a diverse pectin matrix and proteoglycans (extensins and arabinogalactan proteins) [14]. The extracellular XyG polymer is made of β-1,4-linked D-glucose with functional glycosyl side-chains. In *Arabidopsis thaliana*, typically 75% of the glucose units are substituted with a xylosyl residue, which can be further substituted either with galactose alone or a galactose moiety decorated with a fucose and/or an *O-acetyl* group (overview in Figure S1) [15,16]. The acid growth theory proposes that an auxin-dependent increase in plasma membrane proton pump activity triggers rapid cell wall acidification [17]. The decrease of extracellular pH initiates a cascade of events, including activation of expansins, which dissociate XyG-cellulose networks and consequently promote cell wall loosening [18,19]. However, the complexity of the cell wall and also the concentration- and tissue-dependent effects of auxin question the universal validity of a single growth mechanism (e.g., [7,20]). 

Interestingly, a yet unknown cell wall sensing mechanism perceives defects in the cell wall mechanics, such as the loss of XyG synthesis, and provides an AUXIN RESPONSE FACTOR 2 (ARF2)-dependent negative feedback on intercellular auxin transport in apical hooks [21]. Conversely, several studies have shown that auxin signaling affects various XyG-related genes suggesting an effect of auxin signaling on XyG-related processes [22,23,24,25,26,27,28,29,30]. However, the contribution of such a potential interplay to differential growth remains unknown. Here we show that growth inducing and repressing conditions reduce and stimulate the molecular complexity of extracellular xyloglucans, respectively. Using genetic, biochemical and imaging approaches, we provide evidence that auxin-dependent growth programs exert a spatial control on XyG structure, namely on the level and types of backbone substitutions, which contributes to gravity induced, differential growth in dark grown hypocotyls.

## 2. Results

### 2.1. Auxin-Induced Cell Expansion Correlates with Spatial Changes in the Structure of Xyloglucans in Pea and Arabidopsis

**Figure 1:**
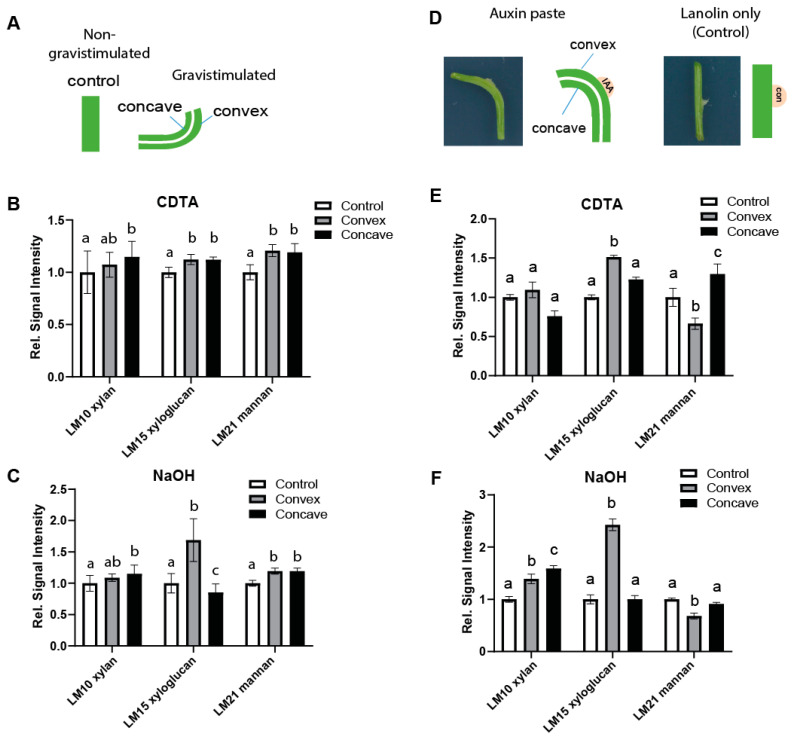
(**A**–**C**) Comprehensive Microarray Polymer Profiling (CoMPP) of differentially elongated stem segments after gravistimulation. The LM15 antibody, specific to the non-galactosylated (XXXG) motif of Xyloglucan (XyG), showed increased epitope detection in longer stem segments. (**A**) Schematic of the experimental design. (**B**,**C**) Quantification of relative changes in the signal intensities with a cyclohexanediaminetetraacetic acid (CDTA) (**B**) and NaOH (**C**) extraction in relation to non-stimulated control (*n* = 10 sectioned pairs, error bars represent SEM). Two-way ANOVA followed by Tukey’s test with *p*-value < 0.05. (**D**–**F**) CoMPP profiling of pea segments after three-day treatment with an indole-3-acetic acid (IAA)-containing lanolin paste. (**D**) Schematic representation of the auxin application assay. (**E**,**F**) Relative quantifications of both CTDA (**E**) and NaOH (**F**) extractions, in comparison to the non-treated control (*n* = 7 independent curvature sections, error bars represent SEM). Two-way ANOVA followed by Tukey’s test. Similar letters in the graphs mark no significant statistical difference. Different letters in the graphs mark significant statistical difference with a *p*-value < 0.05.

In order to study auxin-reliant differential growth, we exposed plants to a gravitropic stimulus, which activates a complex sequence of events ultimately inducing an asymmetric increase of auxin and consequently cellular elongation at the lower side of the shoot [31]. We initially evaluated pea stems, because they provide material in quantities sufficient for immunoglycan profiling and are amendable to local auxin manipulation. We longitudinally dissected gravistimulated stems and separated the longer (more elongated, convex) and shorter (less elongated; concave) sides (Figure 1A). 

On these samples, we performed comprehensive microarray polymer profiling (CoMPP) [32,33], using specific antibodies against different cell wall epitopes [34] (Figure S2A,B). We followed established protocols employing a calcium chelator cyclohexanediaminetetraacetic acid (CDTA) to extract the calcium-linked pectin matrix and to liberate the soluble cell wall fraction. Subsequently, we utilized 4 M NaOH to solubilize hydrogen-bonded hemicelluloses, including XyG, and other more tightly bound cell wall components [32,33]. Notably, the LM15 monoclonal antibody (mAb), generated against non-galactosylated and non-fucosylated xyloglucan fragments with the XXXG motif [35], displayed a notable gravity-induced asymmetry when used against NaOH extracts. In comparison, two other hemicellulose epitopes, such as xylan (LM10 mAb) and mannan (LM21 mAb) (Figure 1A–C), as well as other cell wall epitopes (Figure S2A,B) did not show such a defined differential distribution. This finding is in agreement with earlier studies, which suggested that auxin affects the solubility/properties of XyGs [36,37]. To assess if the altered galactosylation status of XyGs indeed relates to auxin-induced differential elongation, we used local application of auxin (lanolin paste) to pea stems, causing the stem to bend (Figure 1D). In agreement with our gravity experiment, asymmetric application of auxin also induced changes in the abundance of LM15 epitopes at the site of application (Figure 1E,F). This set of data suggests that auxin-mediated growth is associated with less substituted XyGs in pea stems.

**Figure 2:**
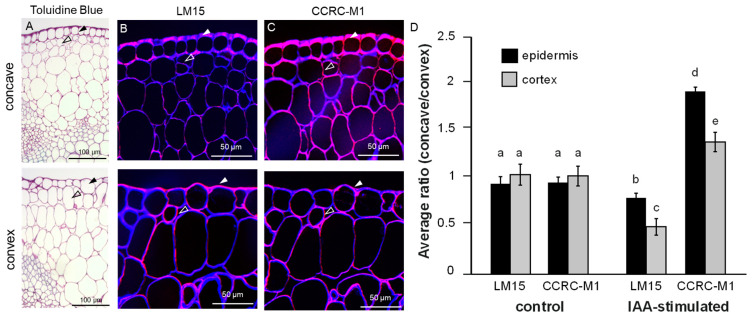
(**A**–**D***)* In situ spatial distribution XyGs in pea sections. (**A**) Toluidine staining of the thin resin section through auxin-modulated pea segment. Close up pictures on the tissue morphology of the concave (shorter) and the convex (longer, auxin-modulated). Note the enlargement of the epidermal (closed arrowhead) and cortical cells (open arrowhead) in the convex site. (**B**–**D**) Immunolocalization of LM15 (**B**) and CCRC-M1 (**C**) epitopes in concave (upper panels) and convex (lower panels) sides of the auxin paste-modulated stem. Images are overlays of the monoclonal antibodies (mAb)-generated signal (red) and the cell wall counterstaining with β-(1,4)-glucan-specific dye Calcofluor White (blue). Note the decrease of CCRC-M1 signal, but significant increase of the LM15 signal in the epidermis and cortex of the convex side. Closed arrowhead: epidermis; open arrowhead: cortex. (**D**) Quantification of the LM15 and CCRC-M1-generated signal ratios between the concave and convex side of the indole-3-acetic acid (IAA)-stimulated stem. Signals of epidermis and cortex were analyzed separately. Two sides of the control stem (lanolin only) showed no statistically significant differences in LM15 and CCRCM1 labeling (*n* > 26), One-way ANOVA with Tukey’s post-hoc test with *p* < 0.05. Error bars represent SEM. Similar letters in the graphs mark no significant statistical difference. Different letters in the graphs mark significant statistical difference with a *p*-value < 0.05.

The detected changes in a CoMPP abundance could hint at spatial alterations in XyG structure or may alternatively relate to altered extractability of XyG moieties. Therefore, we studied the spatial distribution of XyGs in situ using immunolocalization procedures (Figure 2) In addition to LM15, we also used the mAb CCRC-M1, which is specific for α-L-fucosylated xyloglucan. Auxin application induced a more intense LM15 antibody labeling in the convex site, when compared to the concave side of the stem (Figure 2B,D and Figure S3A,B). Conversely, CCRC-M1 mAb labeling showed lower signal in the convex side in comparison to the concave side (Figure 2C,D and Figure S3A,B). In agreement, we also observed a similar, gravity induced asymmetry of CCRC-M1 labeling in dark grown *Arabidopsis* hypocotyls (Figure S4A,B). This set of data suggests that auxin induced cell expansion correlates with spatially defined changes in the molecular structure of XyGs.

### 2.2. Auxin Signaling Defines Complexity of XyG Structures

Next, we genetically repressed nuclear auxin signaling by overexpressing PIN-LIKES (PILS) proteins in *Arabidopsis*. PILS proteins are endoplasmic reticulum (ER) localized auxin transport facilitators that repress nuclear abundance and signaling of auxin, presumably by reducing auxin diffusion into the nucleus [5,6,38,39]. The overexpression of PILS5 reduces auxin signaling and reduces growth in dark grown hypocotyls [5], which correlated with moderate alterations to the monosaccharide composition of wall preparations, showing slightly increased galactose as well as mildly decreased levels of rhamnose and xylose in dark grown hypocotyls (Figure S5A). Alterations in galactose levels may primarily relate to changes in Arabinogalactan proteins (AGPs) and/or pectic polysaccharides, such as galactans, but could also hint at alterations in XyG structure [40]. To specifically investigate the effect of auxin signaling depletion on XyG structure, we next used endoglucanase to generate XyG fragments to be analyzed via oligosaccharide mass profiling (OLIMP) by MALDI-TOF/MS [41] and most prominently observed an increase in fucosylation of XyGs in PILS5 overexpressing lines when compared to Wt (Figure S5B), independently confirming that low auxin signaling rates induce complex XyG structures in *Arabidopsis*.

Altogether, the combined approaches of CoMPP, immunocytochemistry and OLIMP revealed that auxin signaling indeed adjusts XyG structure, possibly providing spatially defined readouts. 

### 2.3. Genetic Modification of XyGs Define Auxin-Dependent Differential Growth

In order to assess whether these structural alterations indeed define auxin-mediated differential growth, we genetically interfered with XyG complexity by targeting the genes encoding for fucosyltransferase *MURUS2* (*MUR2*) and the fucosidase *altered xyloglucan8* (*AXY8*) as well as with the galactosyltransferase *MUR3* and the beta-glactosidase10 (*bGAL10*) in *Arabidopsis*. Initially, we exogenously exposed *mur2* and *axy8* as well as *mur3* and *bgal10* dark grown mutants to auxin (Figures S6A–I and S7A,B). Galactosylation of XyGs appears to have a more pronounced effect on auxin-reliant growth than fucosylation, because hypocotyl expansion of *bgal10*, but not *axy8* mutants, was partially resistant to high exogenous levels of auxin (Figure S7A,B). In agreement, genetic crosses with *bgal10* mutants partially alleviated the overexpression phenotype of auxin-biosynthesis enzyme YUCCA8 (YUC8) (Figure S7C–F). Moreover, we noted that *mur3* mutant alleles displayed an amplified loss of gravitropic growth when exposed to exogenous auxin (Figure S6A,C,D,F). The curved *mur3* dark grown hypocotyl phenotype was reminiscent to seedlings exposed to high levels of auxin (Figure S7A). Accordingly, the *mur3* mutants might display some hypersensitivity to auxin, but on the other hand, the auxin induced repression of hypocotyl expansion was not enhanced when compared to Wt seedlings (Figure S6A,B,D,E). However, this relative assessment could be biased due to the already short hypocotyl phenotype of untreated *mur3* mutants (Figure S6A,B,D,E).

**Figure 3:**
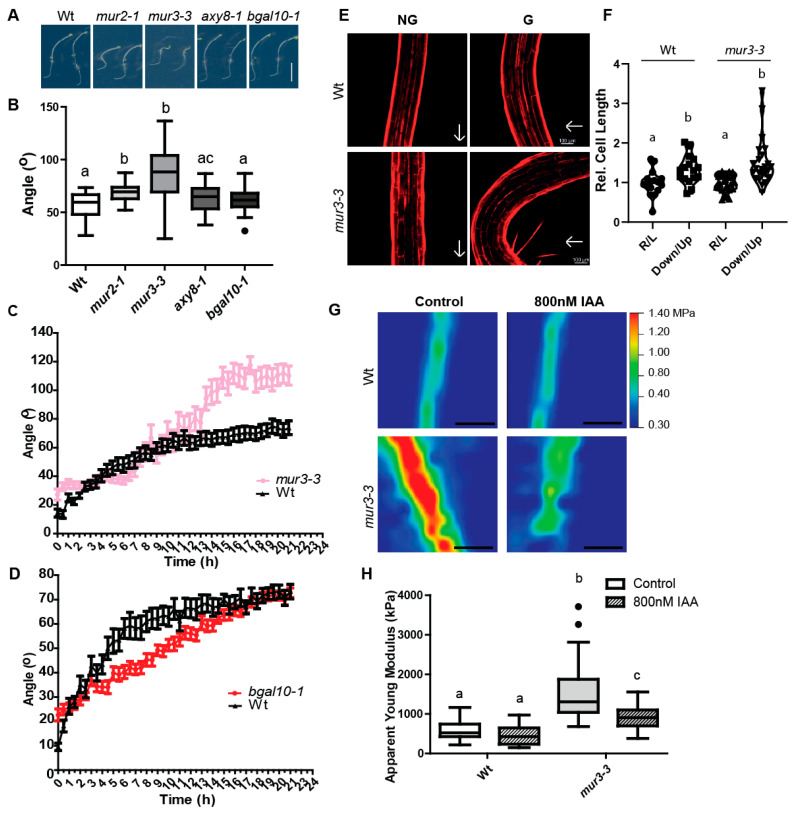
Response to gravistimulation of *murus2-1 (mur2-1)*, *murus3-3 (mur3-3)*, *altered xyloglucan8 (axy8-1)* and *beta-galactosidase10-1 (bgal10-1)*. (**A**,**B**) Five-day-old dark-grown hypocotyls were challenged with a 90° angle change in growth orientation and the end point angle between the apex of the hypocotyl and gravity vector was measured 24 h later. (**A**) Representative images of the angular hypocotyl growth after 24 h. (**B**) Quantification of the end point angle. Tukey box-plot. One-way ANOVA followed by Tukey’s test. *p*-value < 0.05. Scale bar = 5 mm (*n* = 3 biological replicates with 20–30 angle measurements each). (**C**,**D**) Growth kinetics of *mur3-3* (**C**) and *bgal10-1.* (**D**) Five-day-old dark-grown hypocotyls were challenged with a 90° angle change in growth orientation and placed in an infrared-based dark-imaging box where their growth was recorded. The angle reached every 30 min was quantified. Non-linear fit to a one-phase association curve. K values for each curve were compared. *p*-value < 0.05. Data are mean ± SEM (*n* = 3 biological replicates with 12–28 seedlings each). (**E**,**F**) Cell elongation of Wt and *mur3-3* after gravistimulation. Five-day old etiolated hypocotyls were challenged with a 90° angle change in orientation and left overnight, and then stained with propidium iodide. (**E**) Representative images. Scale bar = 100 µm. (**F**) Quantification of relative cell length. Between 2–3 cells from the cortex region were measured on the right (R) side and left (L) side of non-gravistimulated (NG) hypocotyls, or from the downwards side (Down) or upwards side (Up) of gravistimulated (G) hypocotyls. The average of relative cell length between right and left (R/L), and down and up (Down/Up) is reported. Data are mean ± SD (*n* = 3 biological replicates of cell length ratios corresponding to 20–22 seedlings). Kruskal–Wallis one-way ANOVA followed by Dunnett’s test; *p*-value < 0.05. (**G**,**H**) Atomic force microscopy (AFM) analysis of *mur3-3*. Dark-grown hypocotyls of *mur3-3* and Wt Col-0 were grown for three days on 800 nM indole-3-acetic acid (IAA) or dimethyl sulfoxide [DMSO, (Control)]. (**G**) Representative apparent Young’s modulus heat maps of one cell wall region perpendicular to the indentation axis and parallel to the growth axis (periclinal cell wall). Scale bar = 5 µm. (**H**) Quantification of apparent Young’s modulus in kilopascal (kPa). Tukey box-plot. One-Way ANOVA followed by Tukey’s test; *p*-value < 0.05 (*n* = 3 biological replicates with 30–40 AFM scans). Similar letters in the graphs mark no significant statistical difference. Different letters in the graphs mark significant statistical difference with a *p*-value < 0.05.

In order to evaluate the importance of XyGs complexity for an endogenous auxin response, we examined the gravitropic responses in these XyG-related mutants. The *mur3* alleles and *mur2-1* mutant hypocotyls showed gravitropic defects when challenged with a 90° angle change in growth orientation (Figure 3A,B and Figure S8A,B). However, compared to *mur3-3* and *mur3-7*, gravitropic growth of *mur2-1* mutants was markedly less affected. The expression of pMUR3:MUR3-mScarlet in *mur3-3* alleviated the growth reduction and complemented the gravitropic defects (Figure S9A–E), additionally confirming that MUR3 has a defining role in gravitropic hypocotyl growth. On the other hand, *axy8* and *bgal10* mutants were not distinguishable from wild type seedlings in this gravitropic end point measurement experiment (Figure 3A,B). To further asses this phenotype in detail, we performed infrared-based growth kinetics measurements of gravitropic dark grown hypocotyls. We observed a hyper-bending response of *mur3* mutants (Figure 3C), which relates to enhanced differential cellular elongation rates at the upper and lower hypocotyl flanks (Figure 3E,F). Accordingly, we conclude that the auxin-dependent, gravity-induced growth is accelerated in *mur3* mutants. Conversely to the hyperbending phenotype of *mur3*, the growth kinetics of *bgal10-1* mutants initially showed slower gravitropic bending when compared to Wt (Figure 3D), which also agrees with its partial resistance to auxin (Figure S7A–F). 

A family of xylosyltransferases (XXT) are responsible for the bulk of the xylosylation of the glucan backbone (Figure S1). Accordingly, XyGs are not detectable in *xxt1 xxt2* mutants [42], which also correlated with defects in gravitropic hypocotyl growth (Figure S10A–E). When compared to the loss of the fucosylation machinery or even the complete lack of XyGs (Figure S10A–E), the MUR3/bGAL10 dependent galactosylation of XyGs appears to have a particular developmental importance for auxin-dependent gravitropic hypocotyl growth.

Altogether, these results show that genetic modification of XyG complexity distinctly defines auxin-dependent differential growth rates during gravitropic hypocotyl growth.

### 2.4. MUR3 Defines the Auxin Effect on Cell Wall Mechanics

Our results suggest that auxin defines the decorations of XyG sidechains and that this fine-tuning takes place in a spatially restricted manner, contributing to differential growth control. It is currently unknown how precisely the alterations in XyG structure impact on cell wall mechanical properties. To further assess the contribution of MUR3 to local cell wall mechanics, we used atomic force microscopy (AFM) to quantify cell wall properties in epidermal hypocotyl cells of full knockout mutant alleles, such as *mur3-3* and *mur3-7* [43]. The apparent Young’s modulus obtained for Wt dark grown hypocotyl agreed with the previous published data [44,45,46]. However, the cell walls of untreated dark grown *mur3-3* and *mur3-7* mutant hypocotyls were much stiffer when compared to Wt (Figure 3G,H and Figure S8C,D). This observation correlates with an overall reduction in cell size and hypocotyl growth in *mur3* mutants (Figure 3E,F and Figure S6A,B,D,E). 

Notably, exogenously applied auxin [indole-3-acetic acid (IAA), 800 Nm] induced a stronger softening of *mur3-3* and *mur3-7* mutant cell walls when compared to Wt (Figure 3G,H and Figure S8C,D). Accordingly, we conclude that MUR3 defines the auxin impact on wall mechanics, which also agrees with the enhanced gravitropic growth of *mur3* mutants. On the other hand, the here observed softening of *mur3* mutant cell walls was not sufficient to induce organ growth (Figure S6A,B,D,E), suggesting additional growth inhibitory effects when auxin is exogenously applied. 

### 2.5. The Growth Mechanism Defines the Complexity of XyGs

**Figure 4:**
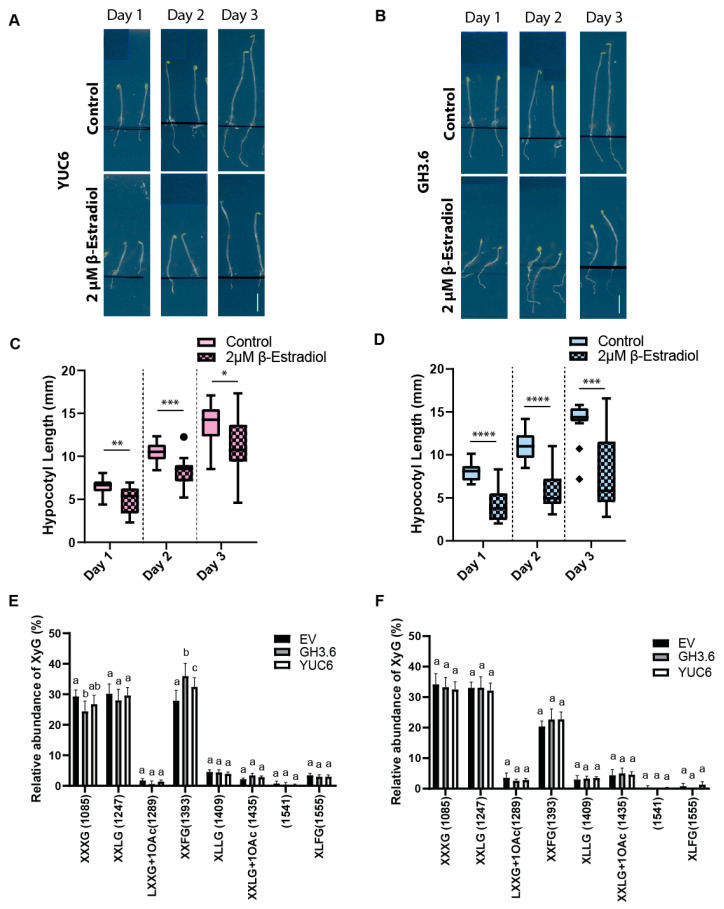
(**A**–**D**) Hypocotyl growth phenotype of estradiol inducible YUCCA6 (YUC6), GRETCHEN-HAGEN3.6 (GH3.6) and empty vector control (EV). Three-day-old seedlings were transferred onto 2 μM estradiol or control plates and left for one, two or three days. (**A**,**B**) Representative images of YUC6 (**A**), GH3.6 (**B**) on control media or estradiol-containing media after one, two or three days of induction. Scale bar = five mm. Please note that distracting labels on the scanned plate were removed by covering them with blue boxes (outlined by light blue lines). (**C**,**D**) Quantification of hypocotyl length (mean ± SD) of YUC6 (**C**) and GH3.6 (**D**). *t*-test with *p*-value < 0.05. (*n* = 3 biological replicates with 5–10 seedlings each). (**E**,**F**) Oligosaccharide mass profiling (OLIMP) on upper (growing part) (**E**) and lower (non-growing part). (**F**) sections of hypocotyls of inducible GH3.6, YUC6 and empty vector control (EV). All lines were induced for three hours with 10 µM ß-estradiol. Data are mean ± SD (*n* = 4 biological replicates). *: *p*-value ≤ 0.05, **: *p*-value ≤ 0.01, ***: *p*-value ≤ 0.001, ****: *p*-value ≤ 0.0001. Similar letters in the graphs mark no significant statistical difference. Different letters in the graphs mark significant statistical difference with a *p*-value < 0.05. XyG: Xyloglucan. XyG sidechains ’nomenclature: G (Glucose), X (Glucose + Xylose), L (Glucose + Xylose + Galactose), F (Glucose + Xylose + Galactose + Fucose), Ac: Acetyl group.

Next, we aimed to investigate whether relative changes in auxin abundance or the auxin-reliant growth programs define the complexity of XyGs. When seedlings are grown in darkness, cotyledon-derived auxin stimulates rapid hypocotyl elongation, while suboptimal auxin levels (whether increased or decreased) reduce its expansion [9,47]. For that purpose, we used constructs for estradiol-inducible auxin-biosynthesis enzyme YUCCA6 (YUC6) [48] and auxin-conjugating enzyme GRETCHEN-HAGEN3.6 (GH3.6) [49]. In agreement, estradiol-induced overexpression of YUC6 or GH3.6 have distinct effects on nuclear auxin signaling output markers, such as *AUXIN/INDOLE-3-ACETIC19* (*IAA19*) and *SMALL AUXIN UP RNA77* (*SAUR77*) (Figure S11A). However, both lines inhibited hypocotyl expansion when transferred to estradiol containing plates (Figure 4A–D). 

Already after 3 h of induction, YUC6 increased endogenous IAA levels in dark grown hypocotyls (Figure S11B). On the other hand, only a mild, statistically non-significant, reduction in auxin levels was detected after short term GH3.6 induction (Figure S11B). In order to further assess the biological activity of the lines, we subjected both lines to RNA sequencing. Compared to the empty vector control, we found 2177 and 1909 differentially expressed genes (DEG) after 3 h induction of YUC6 and GH3.6, respectively (Dataset S1). The overlapping genes clustered in four categories, displaying (I) up- or (II) down-regulation in both as well as (III) up- and down- or (IV) down- and up- in YUC6 and GH3.6 induced dark grown hypocotyls, respectively (Figure S11C,D; Dataset S1). We identified 102 genes that showed inverse (category III and IV) regulation after induced YUC6 and GH3.6 overexpression. The gene ontology (GO)-term analysis (Dataset S2) of these DEGs (Dataset S1) showed enrichment for auxin- and cell wall-related pathways, confirming the distinct molecular effects of YUC6 and GH3.6 induction on auxin signaling in dark grown hypocotyls. Furthermore, we observed a reproducible, but rather mild upregulation of the *MUR3* transcript after YUC6 induction (Figure S12A,B). In agreement, we also detected an up-regulation of MUR3-mScarlet protein levels upon an exogenous application of auxin (Figure S12C,D). Accordingly, a transcriptional input of auxin signaling could contribute to the regulation of XyGs. In agreement, the ectopic induction of auxin biosynthesis enzyme YUC6 and the auxin amido synthetase GH3.6 have in part opposing effects on the transcription of XyG biosynthesis genes (Figure S12A).

Next, we made use of the distinct molecular, but overlapping effects of YUC6 and GH3.6 on hypocotyl growth and addressed whether adjustments in cellular auxin levels or the auxin-mediated growth context may correlate with alteration in XyG branching. Performing OLIMP after short term (3 h) application of estradiol, we observed YUC6- and GH3.6-induced increase in fucosylation of XyGs in the upper, expanding part of dark grown hypocotyls when compared to empty vector controls (Figure 4E). On the other hand, the lower part of the dark grown hypocotyls, which is not actively growing or shows at least strongly reduced expansion rates [50,51], did not display the YUC6 and GH3-induced fucosylation of XyGs (Figure 4F). 

Accordingly, we conclude that rather than the relative change in auxin abundance, it is the underlying growth program that defines the complexity of XyGs.

## 3. Discussion

Based on our set of data, we propose that auxin signaling output and its underlying growth programs define XyG backbone substitution. We moreover propose that higher and lower structural complexity of XyG contribute to repression and induction of tissue expansion, respectively (Figure S13). Considering that MUR3 modulates the auxin effect on cell wall mechanics, we hypothesize that spatially restricted impact on XyG composition contributes to cell wall mechanics for differential growth. 

It needs to be seen how precisely auxin-related growth programs molecularly define XyG structure. Notably, our RNAseq data hinted at a transcriptional input of auxin signaling. The induction of YUC6 and GH3.6 initiate opposing transcriptional effects, but both cause overlapping effects on XyG structure and plant growth. Accordingly, we assume that transcriptional auxin responses cannot fully recapitulate the observed molecular alterations. It is hence likely that indirect and/or posttranslational mechanisms also contribute to the modifications of XyGs. Phosphorylation of CELLULOSE SYNTHASE A (CESA) is a known regulation of cellulose biosynthesis [52,53]. Considering the ultrafast auxin-mediated protein phosphorylation response [54] a similar post-translational regulation could be envisioned to directly or indirectly impact on XyG-related enzymes.

Besides the mechanical contribution to cell expansion, the cell wall composition in turn is also sensed and provides a feedback signaling to cellular functions [55]. In apical hooks, *xxt1 xxt2* deficiency exerts a negative feedback on the transcription of auxin transport components, abolishing auxin maxima formation and, hence, differential growth [21]. In contrast to apical hook development, we did not find evidences that this feedback mechanism disrupts asymmetric hypocotyl expansion. In contrast to apical hooks, auxin-reliant differential growth in gravitropic hypocotyls is on the contrary enhanced in *xxt1 xxt2* deficient mutants. These insights suggest a distinct, tissue-specific mode of action for growth control in apical hooks and during gravitropic hypocotyl growth. This is also reminiscent to tissue specific auxin perception mechanisms, because hypocotyl expansion requires canonical TIR1/AFBs transcriptional responses [9], but TMK1-mediated, non-genomic auxin responses contribute to growth repression in apical hooks [11]. On the other hand, AUXIN SIGNALING F-BOX 1 (AFB1) also defines fast, non-transcriptional responses [56].

It remains to be seen how precisely the XyG structure contributes to auxin-reliant wall mechanics for differential growth control in gravitropic hypocotyls. Tissue biophysical measurements using AFM did not show altered mechanical properties in shoot apical meristems of *xxt1 xxt2* mutants when compared to Wt, which is likely due to compensatory mechanisms in this tissue [57]. Our AFM data set shows on the contrary that *mur3* knock out mutants display stiffer cell walls. On the other hand, a close to 50% reduction in tensile strength was detected for hypocotyls of a partial loss-of-function allele *mur3-1* in which MUR3 protein level is reduced [58]. The time-dependent extension analysis (tensile strength) relates to longitudinal forces along an entire organ and hence may not strictly correlate with AFM-indentation-based cellular measurements in the epidermis. These two methods may even show the contribution of different cell wall polymers to distinct cell wall parameters [59]. 

Alterations in XyG structure presumably affect the hydrogen bonding of XyGs with cellulose microfibrils and/or other cell wall polymers, affecting its pH-dependent interactions [60,61]. Accordingly, the interaction of XyGs with other cell wall components may also relate to the observed differences in AFM and tensile strength analysis of *mur3* mutants [58]. In agreement with a complex interaction of distinct cell wall components, arabinoxylans and pectins can compensate for the mechanical load in XyG-deficient mutants [62]. In addition, the CELLULOSE SYNTHASE-like C (CSLC) proteins contribute to XyG biosynthesis, because the quintuple *cslc456812* mutant completely lacks detectable XyGs and phenocopies the *xxt1 xxt2* double mutant [63].

MUR3-dependent galactosylation appears to have particular importance for plant development [64], but in this context it is largely unknown how the galactosylation status of XyGs impact its interactions with other cell wall components. Here we show that MUR3-dependent galactosylation is also most decisive for its impact on auxin-related growth. However, the absence of detectable XyGs (as seen in *xxt1 xxt2* double mutants) also modulates auxin related growth processes, but to a weaker extent. The ectopic *O*-acetylation of XyGs in *Arabidopsis* alters xylosylation pattern and impairs plant growth [65,66], illustrating the importance of backbone modifications. Our work provides further mechanistic insight into the side chain decoration of XyGs, suggesting that the auxin-mediated, spatial alterations in XyG structure could locally impact on the complex cell wall mechanics, allowing for differential tensions and growth along the organ.

## 4. Materials and Methods

### 4.1. Plant Material

The Wt background for all lines described is Col-0. Lines *murus2-1* (*mur2-1; AT2G03220)* [67], *altered xylogucan8* (*axy8-1; AT4G34260)* [68], *murus 3-3 (mur3-3; AT2G20370;* Salk_141953) and *murus3-7 (mur3-7;* Salk_127057) [43], and *beta-galactosidase10-1* (*bgal10-1)* [69] have been previously described. The *axy8-1* line was courtesy of Markus Pauly, *mur3-3* and *mur3-7* were courtesy of Malcom O’Neill, and *bgal 10-1* was courtesy of Ignacio Zarra. All seeds can be obtained from the Arabidopsis Biological Resource Center (https://abrc.osu.edu/; Columbus, OH, USA). The 35s:PILS5-GFP (PILS5^OE^) line was described in Barbez et al. 2012 [5]. Primers used for genotyping are listed on Table S1.

### 4.2. Growth Conditions

Seeds were sterilized overnight with chlorine gas, and afterwards plated in 0.8% agar, 0.5× Murashige and Skoog (MS), and 1% sucrose medium (MS+). For the majority of the experiments (unless stated otherwise), the plates containing the seeds were stratified for two days at 4 °C, and then exposed to cool-white light (140 µmol·m^−2^·s^−1^) for 6–8 h at 21 °C so as to induce germination and subsequently kept in the dark for five days at 21 °C. 

For the auxin treatment experiments, the MS medium was supplemented with 800 nM indole-3-acetic acid (IAA) or less than 0.1% DMSO. The seedlings were placed on this medium and grown as described above.

### 4.3. RNA Extraction and RT-qPCR Analysis

We always used hypocotyl tissue for RNA extractions. For the estradiol-induced assays, a 100 µm pore mesh (Mesh Nitex 03-100/44; VWR, Avantor, Vienna, Austria) was placed on top the MS+ medium, and then the seeds were placed on top of this mesh. The plates were then handled as described above for three days (estradiol treatments). At day three, the plates were uncovered under a green light, so as not to activate any light responses, and the mesh was transferred onto a new plate containing 10 µM β-estradiol, and then kept in the dark for three hours (estradiol), respectively. Tissue was harvested afterwards and total RNA was isolated using the InnuPREP Plant RNA Kit (Analytic Jena), following the manufacturer’s instructions. After RNA extraction, samples were treated with InnuPREP DNase I (Analytic Jena). cDNA was synthesized from 1 µg of RNA using the iSCRIPT cDNA synthesis Kit (Bio-Rad) following manufacturer’s recommendations. We used Takyon qPCR Kit for SYBR assay (Eurogentec) and the RT-PCR was carried out in CFX96 Touch Real-Time PCR Detection System (Bio-Rad). *ACT2* (*AT3G18780*) was used as housekeeping [70,71] unless stated otherwise. For RNAseq validations, gene *AT1G29670* was used as housekeeping, since it was a gene that was stable for all lines and treatments. This gene was selected from the RNAseq data. Primers for all tested genes are listed in Table S1. 

### 4.4. Cloning

Gateway cloning was used to construct *pMDC7_B(pUBQ):GH3.6.* The *GRETCHEN-HAGEN3.6* (*GH3.6)* full-length genomic fragment was amplified by PCR from genomic DNA. Primers are listed in Table S1. The PCR was performed using the high-fidelity DNA polymerase “I proof” (Bio-Rad). The full genomic fragments were cloned into the pDONR221 (Invitrogen) vector using Invitrogen BP-clonase according to manufacturer’s instructions. Coding sequences were transferred from the entry clones to gateway-compatible pMDC7_B(pUBQ) vector [5] using the Invitrogen LR clonase according to manufacturer’s instructions. The resulting construct as well as an empty vector were transformed into Col-0 plants by floral dipping in *Agrobacterium tumefaciens* GV3101 strain liquid cultures.

Gateway cloning was used to construct pMUR3:MUR3-mScarlet. A 1474 bp intergenic region upstream of the starting codon of the *MURUS3* (MUR3) gene was used as the promoter for MUR3. This region was amplified from genomic DNA via PCR using the Q5 NEB high-fidelity polymerase following the manufacturer’s instructions. Primers used are listed in the Table S1. The fragment was cloned on a pJET 1.2 blunt cloning vector following manufacturer’s instructions (Thermo Fisher, Vienna, Austria). The BsaI restriction site present in the fragment was mutated from ggtctc to ggcctc. The full-length genomic *MUR3* fragment was amplified by PCR from genomic DNA with the NEB Q5 high-fidelity polymerase, and subsequently cloned into a pJET 1.2 blunt cloning vector. Primers are listed in the Table S1. The final Goldengate assembly was made in the pGGZ003 destination vector following the protocol described by Lampropoulos et al. [72]. All the other required modules for the assembly were obtained from Addgene (Addgene kit #1000000036) [72]. The resulting construct pMUR3:MUR3-mScarlet was transformed into Wt Col-0 plants following the floral dipping method and selected on MS+ BASTA plates. 

The *mur3-3*/pMUR3:MUR3-mScarlet complementation line was obtained by crossing.

### 4.5. Quantification of Hypocotyl Length and Gravity Index

Seedlings were grown for five days in the dark on vertically orientated plates. After this, the plates were scanned with an Epson Perfection V700 scanner. Hypocotyl length was quantified using FIJI 2.0 software [73].

The Gravity index was calculated as described by [74], where the index is defined as the average cosine of the angle between the gravity vector and the direction of the hypocotyl elongation.

For the ß-Estradiol inducible lines pER8:YUCCA6 [75], pMDC7::GH3.6 and pMDC7 empty vector control (EV), the seeds were first plated onto meshes as described above and after three days, these meshes were transferred onto an MS+ plate containing either dimethyl sulfoxide [DMSO, (control)] or 2 μM ß-Estradiol, and then left for 1, 2 and 3 days. Afterwards, the plates were scanned and the hypocotyl length was measured as detailed above.

### 4.6. Gravi-Stimulation Assays and Quantification

Seedlings were grown for four days and then turned 90° and kept in this position for another 24 h. Afterwards, plates were scanned with an Epson Perfection V700 scanner. We measured the angle that was formed between the apex of the hypocotyl and the gravity vector, using the angle tool of the FIJI software.

For the assay where the seedlings were afterwards stained with propidium iodide (PI), the gravistimulation was overnight.

### 4.7. Real Time Analysis of Gravitropic Response

Seedlings were grown for four days and then turned 90° then placed in this new position in light-sealed box equipped with an infrared light source (880 nm LED) and a spectrum-enhanced camera (EOS035 Canon Rebel T3i) [6]. The angles made between the hypocotyl apex and the gravity vector were measured every 30 min with the angle tool of FIJI. Representative experiments are shown. Gravitropism kinetics were statistically analyzed using a non-linear regression fit to a one-phase association curve [6].

### 4.8. Confocal Imaging and Quantification

Imaging was performed using a Leica TCS SP5 confocal microscope, equipped with HyD detector. The fluorescence signal intensity (Mean Gray Value) was quantified using the LEICA LAS AF Lite software. In all cases, a Region of Interest (ROI) was defined, and the signal intensity was quantified within that region. The same ROI was kept for all analyzed images within said experiment. ROIs used are indicated in the respective figures. Excitation and emission peaks for mScarlet are 561 nm and 607 nm, respectively; and 569 nm and 593 nm for PI.

When PI was used, seedlings were incubated for 30 min (*mur3-3*), and 1 h (Wt) in a PI solution of 0.02 mg/mL. 

### 4.9. RNA-Seq

Three-day old seedlings of pMDC7:GH3.6, pER8::YUC6 [75] and pMDC7 empty vector lines were grown and induced as already described above. After the induction time, hypocotyl tissue was harvested and total RNA was extracted using the RNAeasy Plant Mini Kit (Qiagen) following manufacturer’s instructions. Prior to cDNA synthesis, RNA was treated with the RNase-Free DNase Set (Qiagen) with the manufacturer’s recommendations. 

The RNA libraries and the subsequent sequencing were performed by the Next Generation Sequencing Facility from the Vienna Biocenter (https://www.viennabiocenter.org/vbcf/next-generation-sequencing/; accessed 16 March 2018)The libraries were generated with the NEBNext Ultra II RNA Library Prep Kit for Illumina with poly(A) enrichment. The sequencing was performed on an Illumina HiSeq2500 with 250 bp paired ended fragments.

### 4.10. Bioinformatics Analysis of the RNAseq Data

#### 4.10.1. Data Pre-Processing

Ribosomal RNA reads were removed by mapping the raw reads against the ribosomal transcript sequences using bwa mem [0.7.16a-r1181, [76]]. The paired end reads were extracted from the unmapped reads using bedtools bamToFastq (v2.29.0, [77]) and the Illumina TruSeq adapters were trimmed with cutadapt [78].

#### 4.10.2. Differential Gene Expression Analysis

To determine differential gene expression in the pER8:YUC6 [75] and pMDC7:GH3.6 seedlings compared to the pMDC7 empty vector plant line, we considered the transcript per million (TPM) values estimated with Salmon [v0.9.1, [79]] for the AtRTD2-QUASI transcriptome annotation [80], and used tximport [81] to aggregate the transcript read counts per gene. Differentially expressed genes were obtained with edgeR using the exactTest [82]. Genes were considered differentially expressed for a false discovery rate < 0.05.

#### 4.10.3. GO-Term Analysis

GO-term analysis was performed using the PANTHER Overrepresentation Test (Released 2019.07.11) at http://www.pantherdb.org/ (accessed on 3 December 2018). The enrichment was determined comparing the query list of differentially expressed genes with an *A. thaliana* database using a FISHER test with an FDR < 0.05.

### 4.11. Auxin Measurements

Determination of indole-3-acetic acid (IAA) metabolite levels was performed following the methods described before [83]. As tissue, five-day-old dark-grown hypocotyls of pER8:YUC6 [75], pMDC7::GH3.6 and pMDC7 empty vector control (EV) lines, induced for 3 h on 2 µM β-Estradiol were used. Briefly, 10 mg of tissue were extracted with 1 mL of 50 mM phosphate buffer (pH 7.0) containing 0.1% sodium diethyldithiocarbamate and mixture of stable isotope-labeled auxins standards. A 200 µL portion of each extract was acidified with 1 M HCl to pH 2.7 and purified by in-tip micro solid phase extraction. After evaporation under reduced pressure, samples were analyzed using HPLC system 1260 Infinity II (Agilent Technologies, Santa Clara, CA, USA) equipped with Kinetex C18 column (50 mm × 2.1 mm, 1.7 µm; Phenomenex, Torrance, CA, USA). The LC system was linked to 6495 Triple Quad detector (Agilent Technologies, Santa Clara, CA, USA). All samples were measured in quadruplicate for each genotype.

### 4.12. Atomic Force Measurements and Apparent Young’s Modulus Calculations

The AFM data were collected and analyzed as described elsewhere with minor changes [44]. To examine extracellular matrix properties, we suppressed turgor pressure by immersion of the seedlings in a hypertonic solution (10% mannitol) for at least 20 min before examination. Three-day-old seedlings grown in darkness (in normal AM plate, with or without IAA) were placed in microscopy slides and immobilized using double-glued side tape. We focused on the periclinal cell walls (parallel to growth axis, but perpendicular to the organ surface), and its extracellular matrix. To ensure proper indentations, especially on the regions in the bottom of the dome shape between two adjacent cells, we used cantilevers with long pyramidal tips (14–16 μm of pyramidal height, AppNano ACST-10), with a spring constant of 7.8 N/m. The instrument used was a JPK Nano-Wizard 4.0 and indentations were kept to <10% of cell height (typically indentations of 100–200 nm depth and 500 nN force). Three scan-maps per sample were taken over an intermediate region of the hypocotyls, using a square area of 25 × 25 μm, with 16 × 16 measurements, resulting in 1792 force-indentation experiments per sample. The lateral deflection of the cantilever was monitored and in case of any abnormal increase the entire data set was not used for analysis. The apparent Young’s modulus (EA) for each force-indentation experiment was calculated using the approach curve (to avoid any adhesion interference) with the JPK Data Processing software (JPK Instruments AG, Berlin, Germany), based on the Hertz model adjusted to pyramidal tip geometry. To calculate the average EA for each periclinal wall, the EA was measured over the total length of the extracellular region using masks with Gwyddion 2.45 software (at least 20 points were taken in account). The pixels corresponding to the extracellular matrix were chosen based on the topography map. For topographical reconstructions, the height of each point was determined by the point-of-contact from the force-indentation curve. A total of 12–14 samples were analyzed. A standard t-test was applied to test for differences between genotypes. 

### 4.13. Monosaccharide Composition Analysis

The analysis was performed using four-day-old dark grown hypocotyls on MS half strength supplemented with sucrose. Two grams of this tissue were used to prepare alcohol-insoluble material to be used in the later analysis. For this purpose, hypocotyls were washed twice in four volumes of absolute ethanol for 15 min, then rinsed twice in four volumes of acetone at room temperature for 10 min and left to dry under a fume hood overnight at room temperature. For determining the neutral monosaccharide composition, 10 mg of dried alcohol-insoluble material were hydrolyzed in 2.5 M trifluoroacetic acid for 1 h at 100 °C as described by [84]. The released Glucose was diluted 500 times and subsequently quantified by means of High Performance Anion Exchange Chromatography- pulsed amperometric detection (HPAEC-PAD) chromatography.

### 4.14. Xyloglucan Fingerprinting (Oligosaccharide Mass Profiling (OLIMP))

Using a green light, five-day old dark-grown seedlings were collected and stored in cold ethanol. Four hypocotyls were dissected for each biological repeat (n = 4), and later used for the analysis. After being left overnight at room temperature in ethanol, the ethanol was removed and the hypocotyls were dried at 37 °C for 1 h. Afterwards, 20 µL of 50 mM acetate buffer, pH5.0, containing endoglucanase from *Trichoderma longibrachiatum* (Megazyme, Scotland, UK) were added and left overnight at 37 °C. OLIMP was then carried out as described elsewhere [41] using Super 2,5-dihydroxybenzoic acid (DHB) matrix (9:1 mixture of DHB and 2-hydroxy-5-methoxybenzoic acid; Fluka) instead of DHB. A solution of the XyG fragments after endoglucanase treatment was used to obtain the Matrix assisted laser desorption ionization-time of flight (MALDI-TOF) spectra.

For the pMDC7:GH3.6, pER8:YUC6 and pMDC7 empty vector control (EV), the hypocotyls were grown in the dark for five days on top of 100 µm pore mesh MS+ plates and then the meshes were transferred to 2 µM β-estradiol MS+ plates. The hypocotyls were dissected into upper section and lower section for the analysis. 

### 4.15. Gravi-Stimulation and Auxin-Induced Bending Experiments on Pea

*Pisum sativum* L. cv. Kelvedon Wonder seeds (Frøbutikken, Denmark) were sterilized with hypochlorite solution before germination on wet tissue in a sterilized plastic box in the dark at room temperature for five days. Seedlings with 3–4 cm roots were transferred to soil (Pindstrup Mosebrug A/S, Ryomgaard, Denmark). Plants were grown at 22 °C/20 °C under 16 h/8 h day/night temperature cycle in growth chambers under fluorescent light (200 μmol m^−2^s^−1^) for one week. Plants were laid down horizontally for one week. Curved stems were harvested and dissected longitudinally to separately obtain the longer (more elongated) and shorter (less elongated) sides. Stems at the same internode of the plant growing vertically were collected as control. Fresh samples were weighed and homogenized by TissueLyser (Sigma-Aldrich, Søborg, Denmark) using a metal bead at max speed. Eight sample pairs were used in the Comprehensive Microarray Polymer Profiling (CoMPP) study.

For the auxin-induced bending experiments, pea plants were planted as described above for the gravistimulation experiment. The tops of three-week-old pea plants were removed down to the second internode from the tip. Lanolin paste containing Indole-3-acetic acid (IAA, Sigma-Aldrich, Søborg, Denmark) (10 mg of IAA per 1 g of lanolin) was applied to one side of the second internode. Stems treated with unadulterated lanolin paste were set as controls. Curved or straight stems were harvested after three days of treatment. The three-day period was chosen because it allowed for the full formation of the curvature and the shorter and longer side could be reliably separated. The stems were dissected longitudinally to separate the longer and shorter sides. A set of three stems was used in CoMPP study.

### 4.16. Comprehensive Microarray Polymer Profiling (CoMPP)

CoMPP analysis was carried out as previously described [32]. Tissuelyzed fresh samples were placed in two solvents sequentially, 50 mM cyclohexanediaminetetraacetic acid (CDTA) and 4 M NaOH with 1% (*v*/*v*) NaBH_4_ at 30:1 (μL/mg). After two hours extraction by shaking and 1700 rcf centrifugation, supernatants were printed onto a nitrocellulose membrane in four printing replicates and four dilutions in ratios of 1:2, 1:6, 1:18 and 1:54 (*v*/*v*). The microarray was probed with a range of cell wall component-directed monoclonal antibodies (mAbs) [34] and the intensity of binding was quantified by implementing an individual scaling.

### 4.17. LR Resin Embedding and Sectioning

For *Arabidopsis* experiments, hypocotyl sections of two-day-old seedlings were fixed in 4% paraformaldehyde (PFA) in phosphate buffered saline (PBS) for 45 min afterward washed 4 times with PBS buffer. Samples were dehydrated for 30 min sequentially at 30%, 50%, 70%, 90% and 100% EtOH in PBS. LR white was added to samples dropwise to 10% and incubated at 4 °C for 6 h. Afterwards, the solution was exchanged with 30% LR white in PBS and incubated at 4 °C overnight. The solution was subsequently exchanged with 100% LR white 3 times each with 12 h incubation and polymerized at 60 °C for 36 h. Samples were sectioned at 2.5 µM thickness using a Reichert Ultracut S Wild M3Z microtome mounted with a Diatome Histo Diamond Knife (8.0 mm 45° angle). Sections were placed on glass slides. 

For the pea experiments, the auxin stimulated and control segments of approximately 1 cm of size were fixed in formaldehyde (prepared from 4% paraformaldehyde in PBS by heating). The samples were washed twice with PBS and dehydrated trough ethanol series (30%, 50%, 70%, 96% and absolute ethanol). The LR embedding was performed by incubating the samples first in the 1:1 mixture of LR resin:absolute ethanol (Sigma-Aldrich, Søborg, Denmark) overnight and then in pure resin LR resin overnight. The samples were place to gelatin capsule filled with LR resin, oriented and polymerized in a heat oven at 60 °C overnight. The gelatin capsule was removed, blocks were trimmed and sectioned using Leica ultramicrotome to 1 μm-thick sections which were collected on water drops on charged SuperFrost slides and let to adhere at a heat plate set at 60 °C.

### 4.18. Histological Staining, Immunolocalization and Microscopy

For the Arabidopsis sections, immunolabeling was performed on sections using CCRC-M1 primary antibody raised in mouse (Agrisera, Vännäs, Sweden) [85] with 1:100 dilution with PBS buffer. Secondary antibody anti Cy5 (Jacksson Immunoresearch) was used with dilution of 1:200. Images were taken using Carl Zeiss LSM780 using 40× magnification (Zeiss C-Apochromat 40×/1.2W Corr M27). Cy5 was excited at 633 nM.

For the pea sections, the overall morphology was studied using staining with 1% Toluidine Blue in water for approximately 5 min. After washing the sections twice with water, the sections were observed under light microscope Olympus BX41 and the pictures were recorded with the digital camera. For immunolocalization, the circular regions surrounding the adhered sections were marked with PapPen (Agar Scientific, Vedbaek, Denmark). The sections were blocked with approximately 100 μL of the 5% milk powder/PBS, followed by a 1 h incubation with the mixture of CCRC-M1 and LM15 primary antibody in the blocking solution at 1:10 dilution each. After washing three times with PBS, incubation with the mixture of anti-rat or anti-mouse secondary antibody conjugated to AlexaFluor 555 or Alexafluor 488, respectively, diluted at 1:300 in 5% milk powder/PBS (Thermo Fisher, Roskilde, Denmark), washing twice with PBS, incubation for 10 min with Calcofluor White (Merc, 100 μg/mL final concentration) and one time washing with PBS. The sections were finally mounted in Citifluor (Agar Scientific, Vedbaek, Denmark) and covered with a coverslip. 

The observation was done immediately. The Leica SP5 laser-scanning confocal microscope and LAS X software were used for the acquiring of the subsequent images. The Argon laser (488 nm) was used for the excitation of AlexaFluor488 signal and the HeliumNeon (555 nm) laser was used for the excitation of the AlexaFluor555 signal and 405 nm diode for the Calcofluor White signal. The specificity of the labeling signal was compared to control sections labeled with the mixture of secondary antibodies only. The pictures were processed with GIMP2 software to generate overlays and to adjust brightness and contrast. If not indicated otherwise the reagents and chemicals were purchased from (Sigma-Aldrich, Søborg, Denmark). The signal intensity was quantified using ImageJ software and the statistical difference was assessed by one-way ANOVA. 

### 4.19. Data Analysis

All graphs and statistical analysis were made with GraphPad Prism software, versions 5 and 8. Statistical tests are depicted in the figure legends. All experiments were performed at least three times.

## Data Availability

Project accession number is PRJNA721549. NCBI SRA accession numbers are SRR14226357-SRR14226365.

## Supplementary Materials

The following are available online at https://www.mdpi.com/article/10.3390/ijms22179222/s1.
