# Supplementary material for: Xyloglucan Remodeling Defines Auxin-Dependent Differential Tissue Expansion in Plants
Source: Int J Mol Sci. 2021 Aug 26;22(17):9222. doi: 10.3390/ijms22179222 (PMC8430841; doi:10.3390/ijms22179222)
Supplement: Supplementary file 1 [file ijms-22-09222-s001.zip › SI_Velasquez_IJMS_25-08-21.pdf]

International Journal of *Molecular Sciences*

MDPI

# Xyloglucan Remodeling Defines Auxin-dependent Differential Tissue Expansion in Plants

**Silvia Melina Velasquez [1,*], Xiaoyuan Guo [2,‡], Marçal Gallemi [3,‡], Bibek Aryal [4,‡], Peter Venhuizen [1], Elke Barbez [1,5], Kai Alexander Dünser [1], Martin Darino [1,†], Aleš Pěnčík [6], Ondřej Novák [4,6], Maria Kalyna [1], Gregory Mouille [7] , Eva Benková [3], Rishikesh P. Bhalerao [4], Jozef Mravec [2] and Jürgen Kleine-Vehn [5,8,*]**

1 Department of Applied Genetics and Cell Biology, University of Natural Resources and Life Sciences Vienna (BOKU), Muthgasse 18, Vienna 1190, Austria; peter.venhuizen@boku.ac.at (P.V.); elkebarbez@gmail.com (E.B.); kai.duenser@boku.ac.at (K.A.D.); martin.a.darino@gmail.com (M.D.); mariya.kalyna@boku.ac.at (M.K.)

2 Department of Plant and Environmental Sciences, University of Copenhagen, Thorvaldsensvej 40, DK-1871 Frederiksberg C, Denmark; yuanquane@gmail.com (X.G.); mravec@plen.ku.dk (J.M.)

3 Institute of Science and Technology Austria, Klosterneuburg 3400, Austria; marcal.gallemi@ist.ac.at (M.G.); eva.benkova@ist.ac.at (E.B.)

4 Department of Forest Genetics and Plant Physiology, Umeå Plant Science Centre, Swedish University of Agricultural Sciences, SE-901 87 Umeå, Sweden; Bibek.aryal@slu.se (B.A.); ondrej.novak@upol.cz (O.N.); Rishi.Bhalerao@slu.se (R.P.B.)

5 Faculty of Biology, Department of Molecular Plant Physiology (MoPP), University of Freiburg, 79104 Freiburg, Germany

6 Laboratory of Growth Regulators, Faculty of Science, Palacký University and Institute of Experimental Botany, The Czech Academy of Sciences, Šlechtitelů 27, 78371 Olomouc, Czech Republic; alespencik@seznam.cz

7 Institut Jean-Pierre Bourgin, Institut National de la Recherche Agronomique, AgroParisTech, CNRS, Université Paris-Saclay, RD10, 78026 Versailles, Cedex, France; gregory.mouille@inra.fr

8 Center for Integrative Biological Signalling Studies (CIBSS), University of Freiburg, 79104 Freiburg, Germany

* Correspondence: melina.velasquez@gmail.com (S.M.V.); juergen.kleine-vehn@biologie.uni-freiburg.de (J.K.-V.)

† Current Address: Molecular Plant Pathology, Department of Biointeractions and Crop Protection, Rothamsted Research, Harpenden, Hertfordshire AL5 2JQ, UK.

‡ Equal contributions.

The following Supporting Information is available for this article:

**Fig. S1** Schematic representation of Xyloglucan (XyG) structure and enzymes involved in their biosynthesis and turnover.

**Fig. S2** Comprehensive Microarray Polymer Profiling (CoMPP) of differentially elongated pea stem segments after gravistimulation

**Fig. S3** Immunolabelling with LM15 and CCRC-M1 Antibodies of Control-stimulated pea stems

**Fig. S4** Immunolabelling with CCRC-M1 antibody of *Arabidopsis* longitudinal sections

**Fig. S5** Monosaccharide composition and Oligosaccharide composition of 35s:PILS5-GFP in *Arabidopsis*

International Journal of
*Molecular Sciences*

MDPI

**Fig. S6** Auxin sensitivity of galactosylation and fucosylation deficient xyloglucan mutants of *Arabidopsis*

**Fig. S7** Auxin sensitivity of xyloglucan mutants deficient in fucose and galactose apoplastic remodeling

**Fig. S8** Characterization of *mur3-7* allele

**Fig. S9** Complementation of the gravitropic defects of *mur3-3*

**Fig. S10** Xylosylation of xyloglucans

**Fig. S11** RNAseq of YUC6 and GH3.6 estradiol-inducible lines.

**Fig. S12** Effect of Auxin on the transcriptional regulation and protein expression of MUR3

**Fig. S13** Schematic Model for the auxin effect on XyG composition

**Table S1** List of primers used in this study

**Dataset S1** List of differentially expressed genes from the RNA-seq

**Dataset S2** Gene Ontology-term analysis of the four categories determined from the RNA-seq data

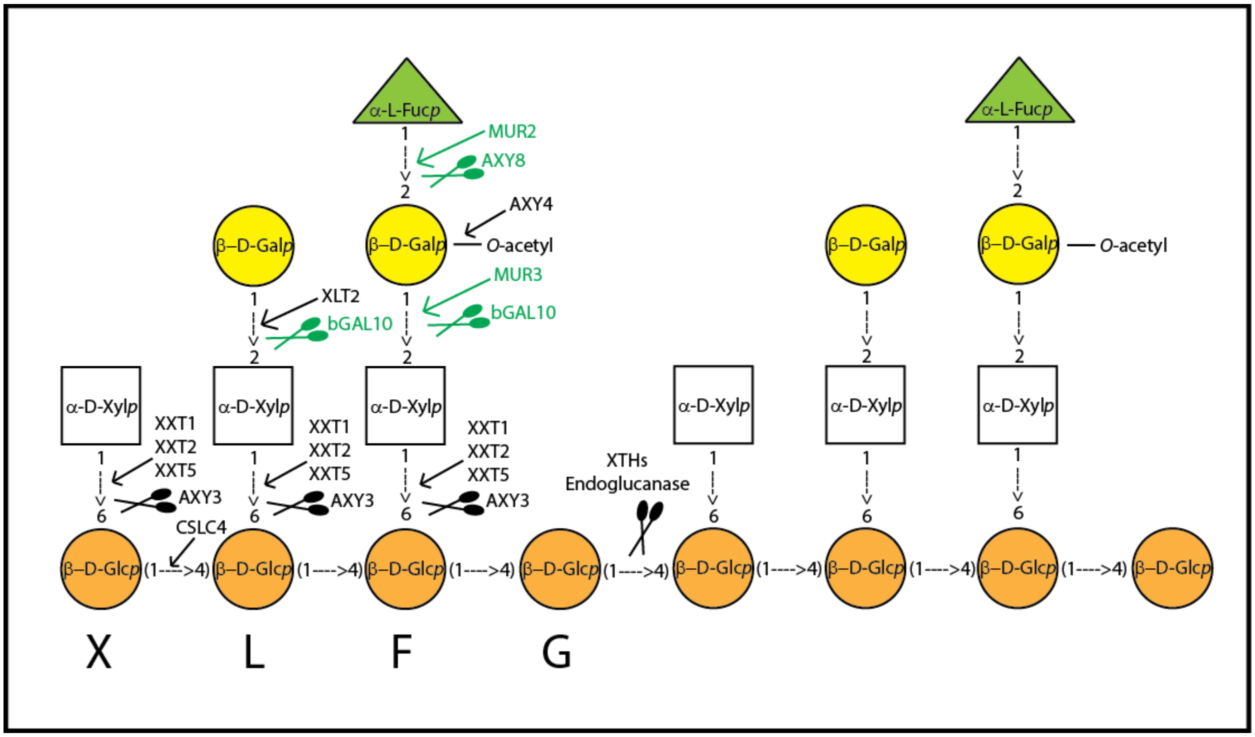


**Figure S1.** Schematic representation of Xyloglucan (XyG) structure and enzymes involved in their biosynthesis and turnover: ALTERED XYLOGLUCAN3 (AXY3), ALTERED XYLOGLUCAN4 (AXY4), ALTERED XYLOGLUCAN8 (AXY8), BETAGALACTOSIDASE10 (BGAL10), CELLULOSE SYNTHASE-LIKE C4 (CSLC4), MURUS2 (MUR2), MURUS3 (MUR3), XYLOGLUCAN L-SIDE CHAIN GALACTOSYLTRANSFERASE POSITION 2 (XLT2), XYLOSYLTRANSFERASE1 (XXT1), XYLOSYLTRANSFERASE2 (XXT2), XYLOSYLTRANSFERASE5 (XXT5), and XYLOGLUCAN ENDO-TRANSGLYCOSYLASE/HYDROLASE (XTH). The arrows and scissors mark the site of action of the different glycosyltransferases and glycosyl hydrolases, respectively. The major XyG biosynthetic and remodelling enzymes discussed in this study are shown in green. XyG nomenclature: the letter G symbols a Glucosyl subunit without any decoration. An X is when a Xylosyl residue is present in the sidechain. L is for a Xylosyl residue further decorated with a Galactose. The letter F depicts a sidechain with Xylose, Galactose and Fucose.

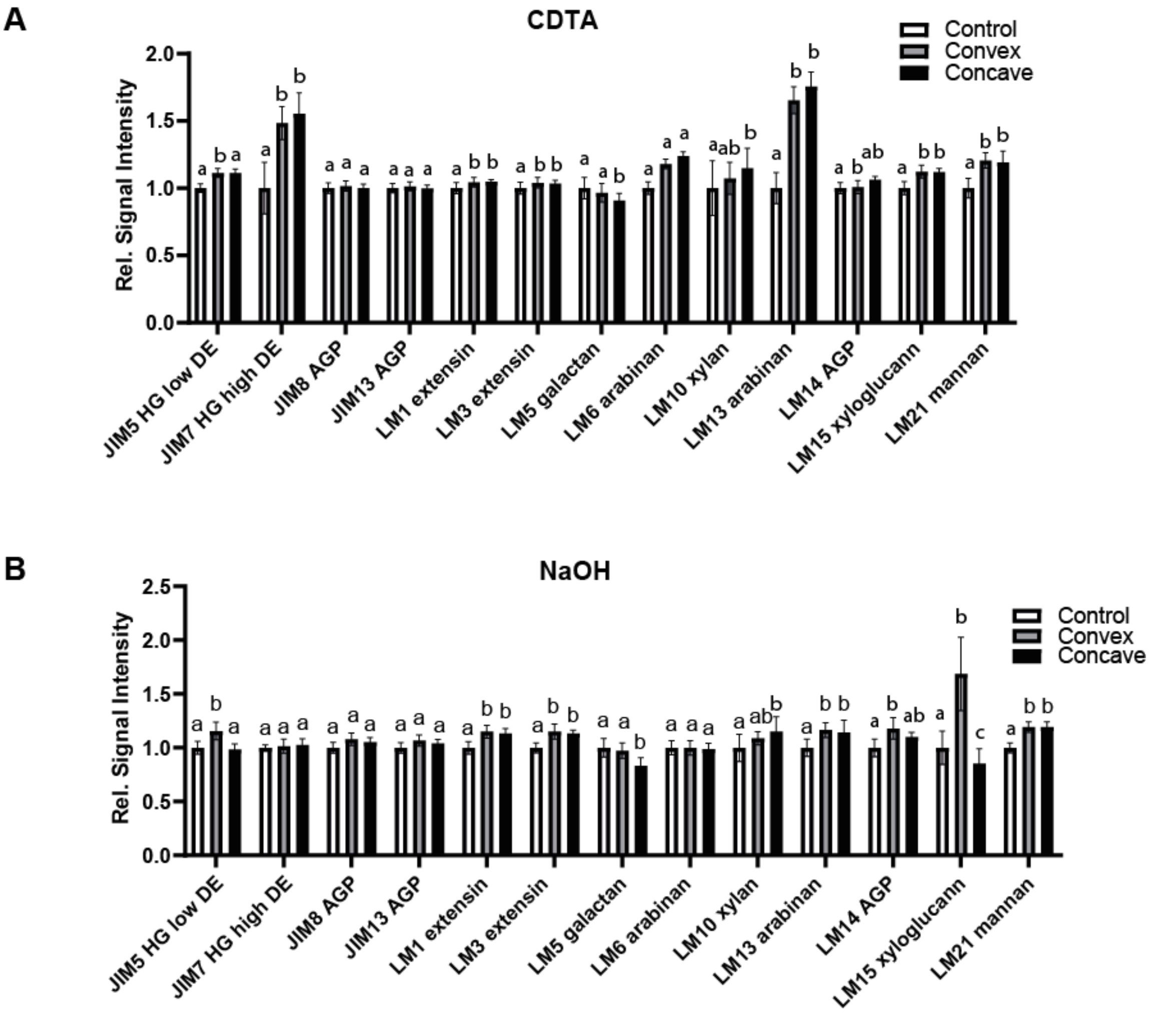


**Figure S2.** Comprehensive Microarray Polymer Profiling (CoMPP) of differentially elongated pea stem segments after gravistimulation. Relative changes in signal intensities in comparison to non-stimulated control set to 1 (n = 3 biological replicates with 9 sections each, error bars represent SEM) with a Cyclohexanediaminetetraacetic acid (CDTA) (**A**) and a subsequent NaOH (**B**) extraction. Two-way ANOVA followed by Tukey's test. Similar letters in the graphs mark no significant statistical difference. Different letters in the graphs mark significant statistical difference with a *p*-value < 0.05.

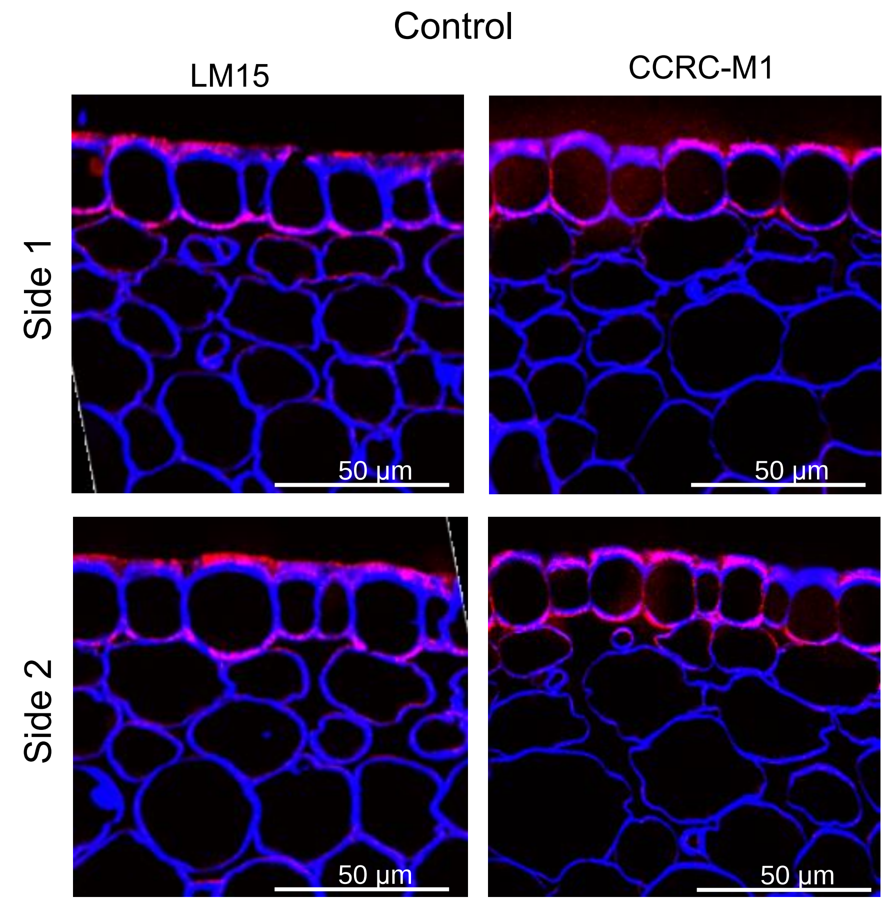


**Figure S3.** Immunolabelling with LM15 and CCRC-M1 Antibodies of Control-stimulated pea stems**.** (**A-B**) Immunolocalisation of LM15 (**A**) and CCRC-M1 (**B**) epitopes in side 1 (upper panels) and side 2 (lower panels) of the two opposite sides of control paste-modulated stem. Images are overlays of the monoclonal antibodies (mAb)-generated signal (red) and the cell wall counterstaining with β-(1,4)-glucan-specific dye Calcofluor White (blue). Note there is no change in the signal intensity of CCRC-M1 and LM15 signal between both mAbs and between the two opposite sides. No statistically significant differences were observed when quantified. Closed arrowhead: epidermis; open arrowhead (cortex).

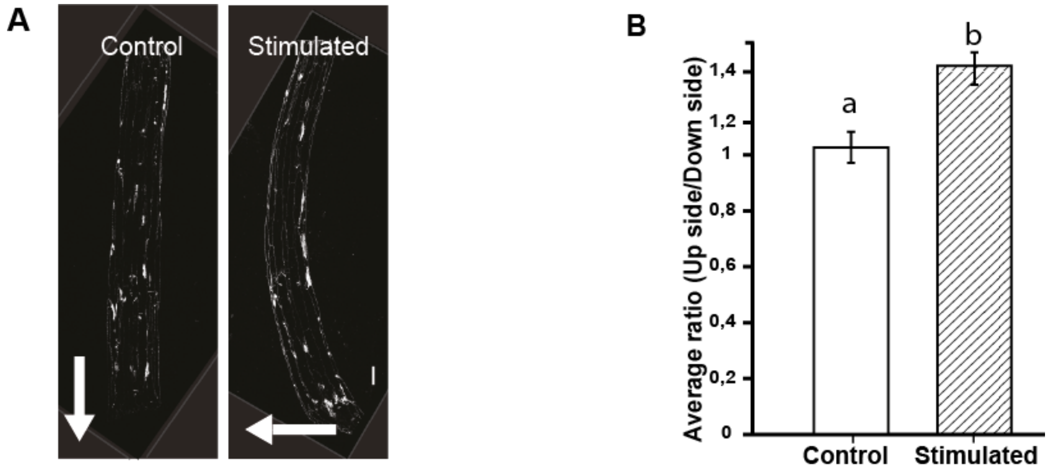


**Figure S4.** (**A-B**) Immunolabelling with CCRC-M1 antibody (specific for fucosylated epitopes of xyloglucans) using longitudinal sections of five-day dark-grown hypocotyls of *Arabidopsis* after gravistimulation and control condition. (**A**) Representative images of sections of non-gravistimulated (Control) and eight hours gravistimulated (Stimulated) dark-grown hypocotyls. Scale bar = 100 µm. White arrows depict the gravity vector. (**B**) Ratio quantification of the mean grey signal at the opposing flanks (e.g. upper and lower) of the hypocotyl section. T-test with *p*-value<0.05 (n = 4 biological replicates). Different letters in the graph mark significant statistical difference with a *p*-value < 0.05.

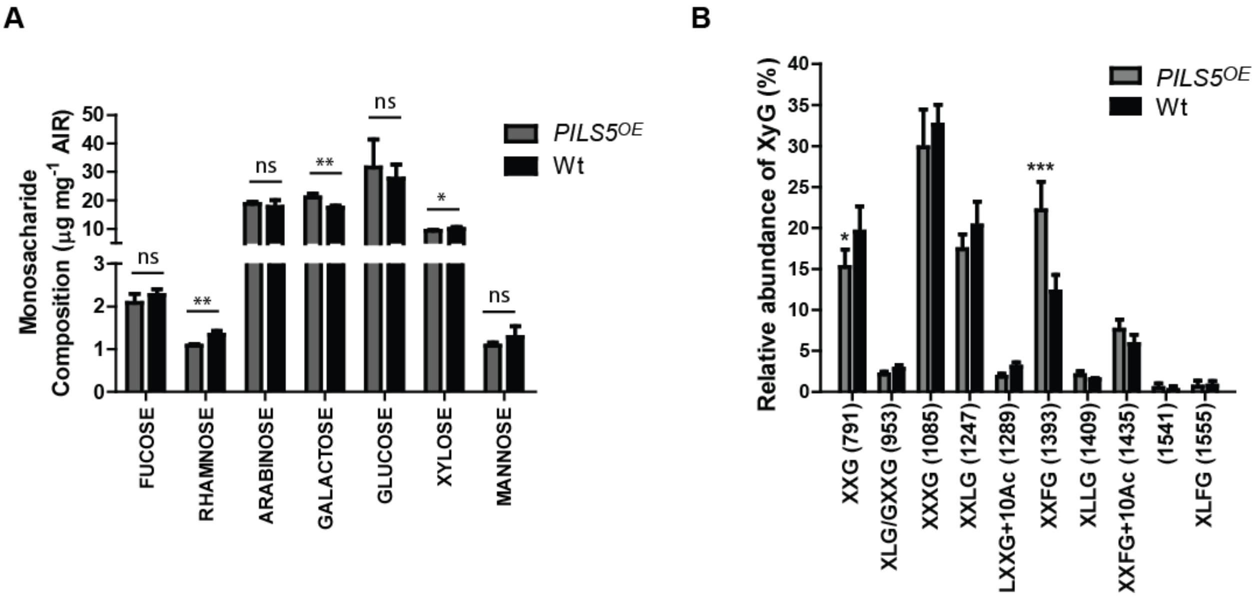


**Figure S5.** (**A**) Monosaccharide composition of wall preparations from etiolated seedlings of *Arabidopsis* (n= 4 biological replicates with 4 technical replicates each). T-test with *p*-value <0.05. (**B**) Oligosaccharide mass profiling (OLIMP) on five-day-old dark-grown hypocotyls of 35s:PILS5-GFP ($PILS5^{OE}$). Data are mean ± SD (n = 3 biological replicates with 3 seedlings). T-test with P-value <0.05. AIR: Alcohol Insoluble Residue; ns: not significant. *: *p*-value≤0.05, **: *p*-value≤0.01, ***: *p*-value≤0.001. XyG: Xyloglucan. XyG sidechains ’nomenclature: G (Glucose), X (Glucose+Xylose), L (Glucose+Xylose+Galactose), F (Glu-cose+Xylose+Galactose+Fucose), Ac: Acetyl group.

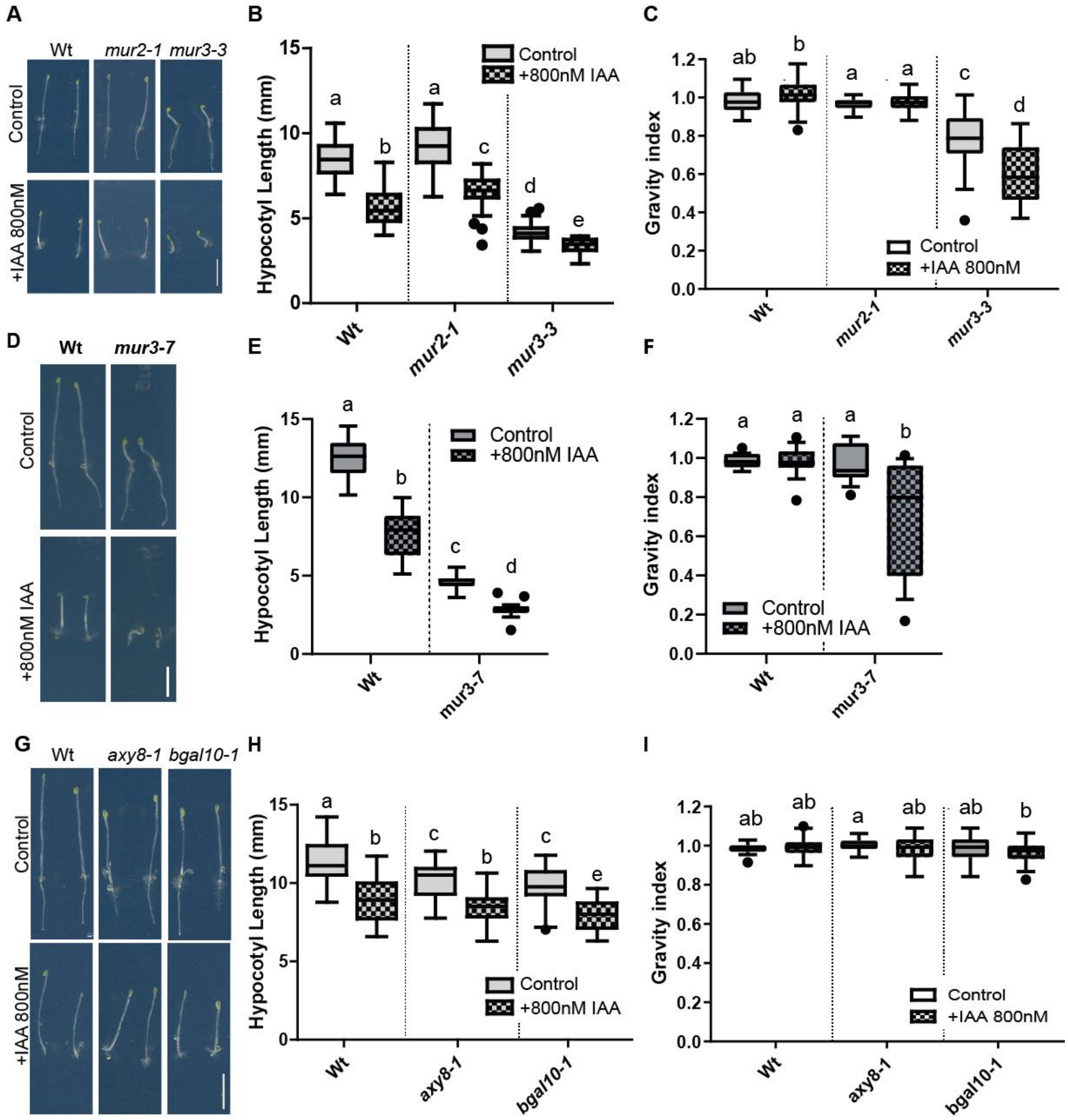


**Figure S6.** Auxin sensitivity of galactosylation and fucosylation deficient xyloglucan mutants of *Arabidopsis*. (**A,D,G**) Mutants of galactosyltransferase *murus3-3* (*mur3-3)* (**A**), *mur3-7* (**D**) and fucosyltransferase *mur2-1* (**A**) and galactosylhydrolase *betagalactosidase10-1 (bgal10-1)* (**G**) and fucosylhydrolase *axy8-1* (**G**) were grown on 800nM indole-3-acetic acid (IAA) or dimethyl sulfoxide [DMSO (Control)] for five days. (**A,D**) Representative images of *mur2-1* (**A**), *mur3-3* (**A**), *mur3-7* (**D**) *axy8-1* (**G**) and *bgal10-1* (**G**). Scale bar = 5mm. (**B, E, H**) Quantification of the hypocotyl length. Quantification for *mur2-1* and *mur3-3* (**B**), *mur3-7* (**E**) and *axy8-1* and *bgal10-1* (**H**). Tukey box-plot (n = 3 biological replicates with 16 – 35 seedlings each). One-Way ANOVA followed by Tukey's test. *p*-value <0.05. (**C,F,I**) Quantification of the Gravity index of *mur3-3* (**C**), *mur2-1* (**C**), *mur3-7* (**F**), *axy8-1* (**I**) and *bgal10-1* (**I**) after 800 nM IAA or DMSO (solvent control) treatment. Tukey box-plot (n = 3 biological replicates with 25-35 seedlings). Two-way ANOVA followed by Bonferroni test with *p*-value <0.05. Similar letters in the graphs mark no significant statistical difference. Different letters in the graphs mark significant statistical difference with a *p*-value < 0.05.

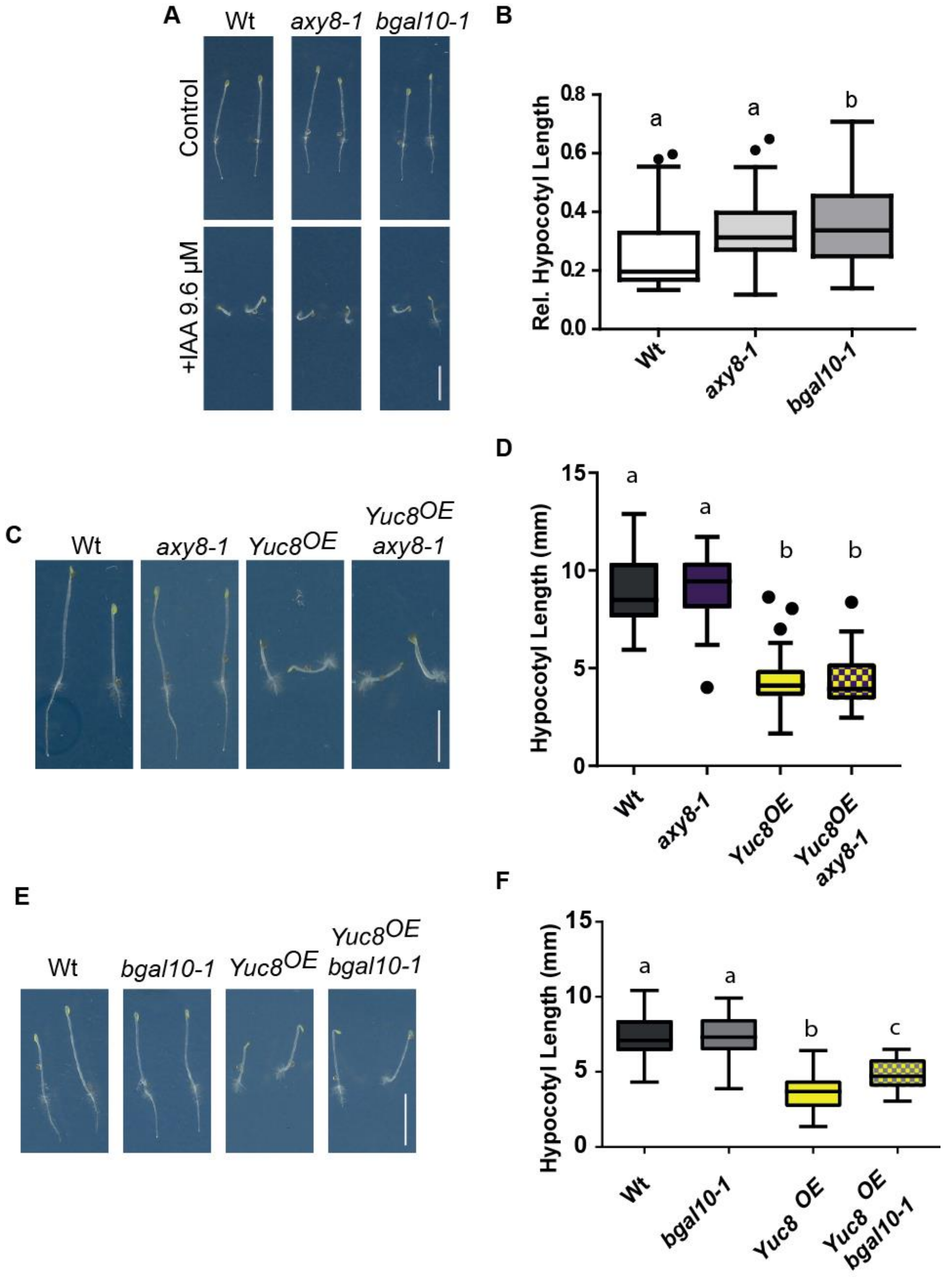


**Figure S7.** Auxin sensitivity of xyloglucan mutants deficient in fucose and galactose apoplastic remodeling. (**A-B**) *altered xyloglucan8-1* (*axy8-1)* and *betagalactosidase10-1* (*bgal10-1)* grown on 9.6 μM indole-3-acetic acid (IAA) or dimethyl sulfoxide [DMSO, (Control)]. (**A**) Representative images. Scale bar = 5mm. (**B**) Quantification of the hypocotyl length relative to the DMSO-treated condition. Tukey box-plot (n = 3 biological replicates with 35-40 seedlings each). One-way ANOVA with *p*-value <0.05. (**C-F**) Genetic interaction between 35s::YUC8 (*Yuc8$^{OE}$*), and *axy8-1 and bgal10-1*. (**C, E**) Representative images of five-day-old dark-grown hypocotyls of *Yuc8$^{OE}$ axy8-1* **(C)** and *Yuc8$^{OE}$ bgal10-1* (**C**). Scale bar =5 mm. (**D, F**) Quantification of the hypocotyl length of *Yuc8$^{OE}$ axy8-1* (**D**) *and Yuc8$^{OE}$ bgal10-1* (**F**). Data are mean ± SD. Tukey box-plot. One-way ANOVA followed by Tukey's test with *p*-value <0.05. (n = 3 biological replicates with 25-40 seedlings each). Similar letters in the graphs mark no significant statistical difference. Different letters in the graphs mark significant statistical difference with a *p*-value < 0.05.

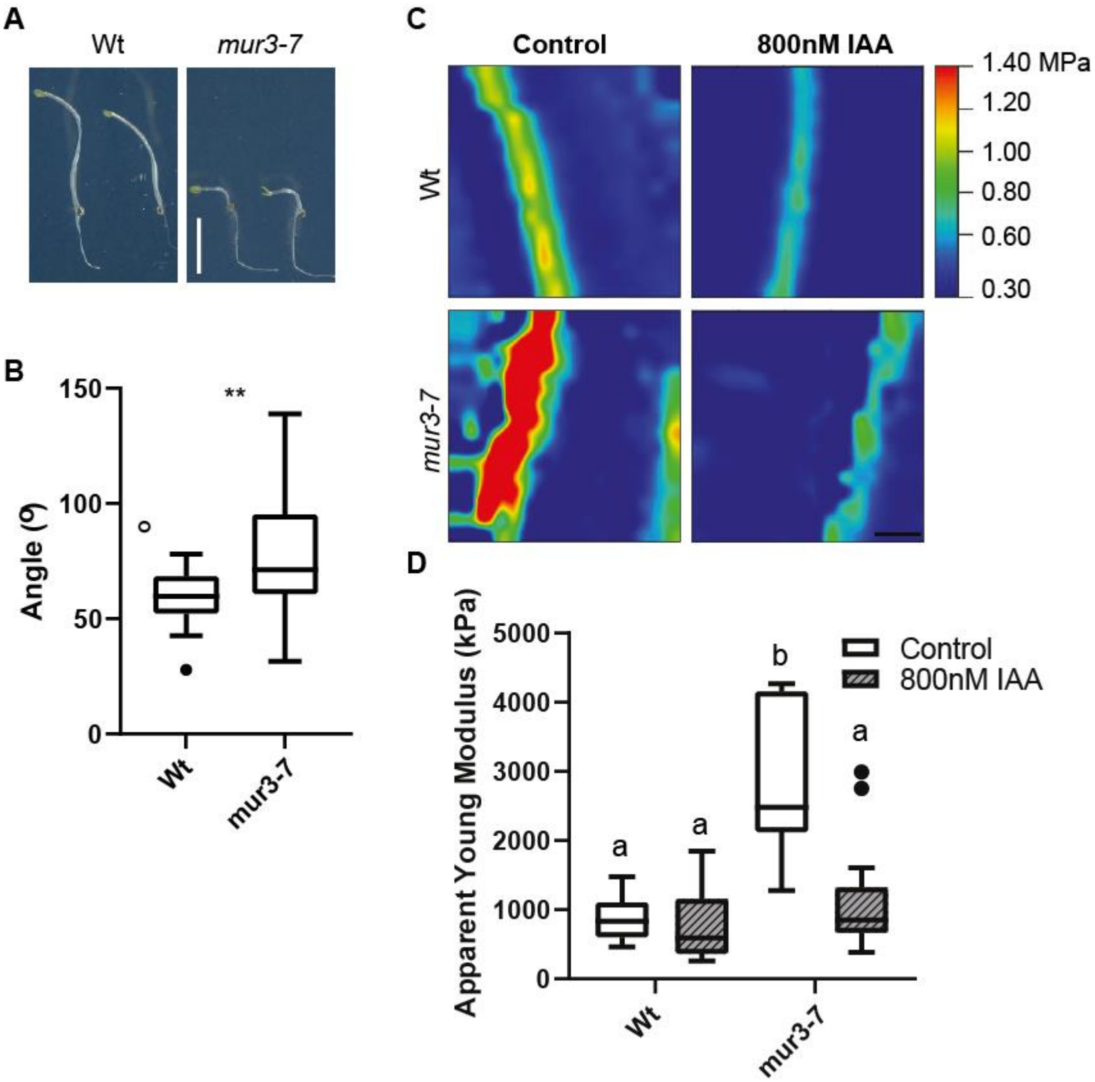


**Figure S8.** Characterization of *mur3-7* allele. (**A-B**) Response to gravistimulation of *murus3-7 (mur3-7)***.** Five-day-old dark-grown hypocotyls were challenged with a 90º angle change in growth orientation and the end point angle between the apex of the hypocotyl and gravity vector was measured 24 h later. (**A**) Representative images of the angular hypocotyl growth after 24 h. (**B**) Quantification of the end point angle. Tukey box-plot. T-test. *p*-value <0.05. Scale bar = 5mm (n = 3 biological replicates with 28-29 angle measurements each). (**C-D**) Atomic force microscopy (AFM) analysis of *mur3-7*. Dark-grown hypocotyls of *mur3-7* and Wt Col-0 were grown for three days on 800nM indole-3-acetic acid (IAA) or dimethyl sulfoxide [DMSO (Control)]. (**C**) Representative apparent Young's modulus heat maps of one cell wall region perpendicular to the indentation axis and parallel to the growth axis (periclinal cell wall). Scale bar = 5µm. (**D**) Quantification of apparent Young's modulus in kPa. Tukey box-plot. One-Way ANOVA Kruskal-Wallis test with *p*-value <0.05 (n = 3 biological replicates with 16-20 AFM scans). **: *p*-value≤0.01. Similar letters in the graphs mark no significant statistical difference. Different letters in the graphs mark significant statistical difference with a *p*-value < 0.05.

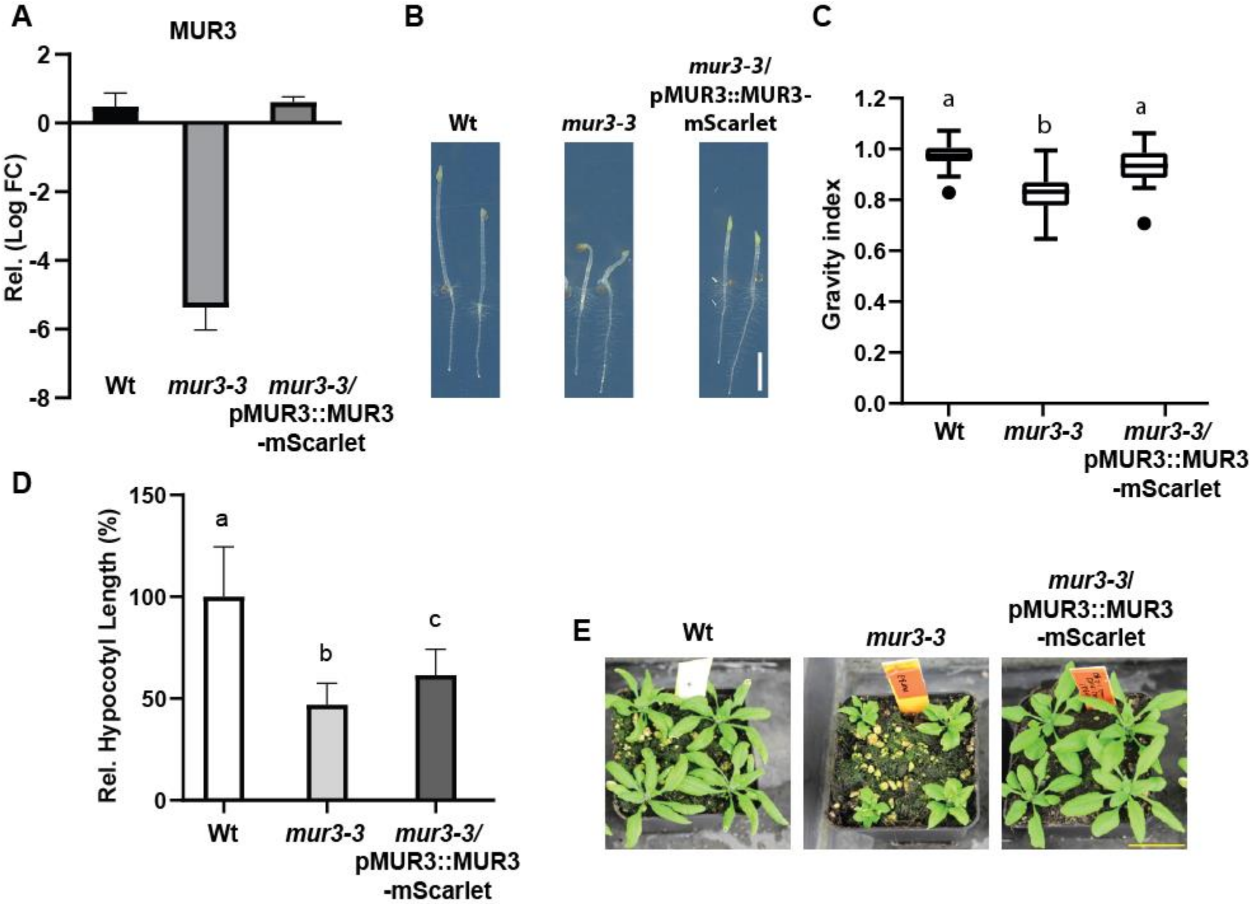


**Figure S9.** Complementation of the gravitropic defects of *mur3-3*. (**A-D**) Five-day-old dark-grown hypocotyls of the *murus3-3* (*mur3-3)* mutant complemented with pMUR3:MUR3-mScarlet. (**A**) RT-qPCR of *MUR3* gene expression. The Log2 fold change (FC) values were made relative to Wt. Data is mean ± SD (n = 3 technical replicates). (**B**) Representative images. Scale bar = 2.5mm. (**C**) Quantification of the Gravity Index. Tukey box-plot (n = 3 biological replicates with 26-30 seedlings each). One-way ANOVA followed by Tukey's test with *p*-value<0.05. (**D**) Quantification of the hypocotyl length relative to the average Wt value represented as percentages. Brown-Forsythe and Welch ANOVA followed by Dunn's test with *p*-value<0.05 (n = 3 biological replicates with 26-30 seedlings each). (**E**) Representative images of four-week old rosettes. Scale bar = 3 cm. Similar letters in the graphs mark no significant statistical difference. Different letters in the graphs mark significant statistical difference with a *p*-value < 0.05.

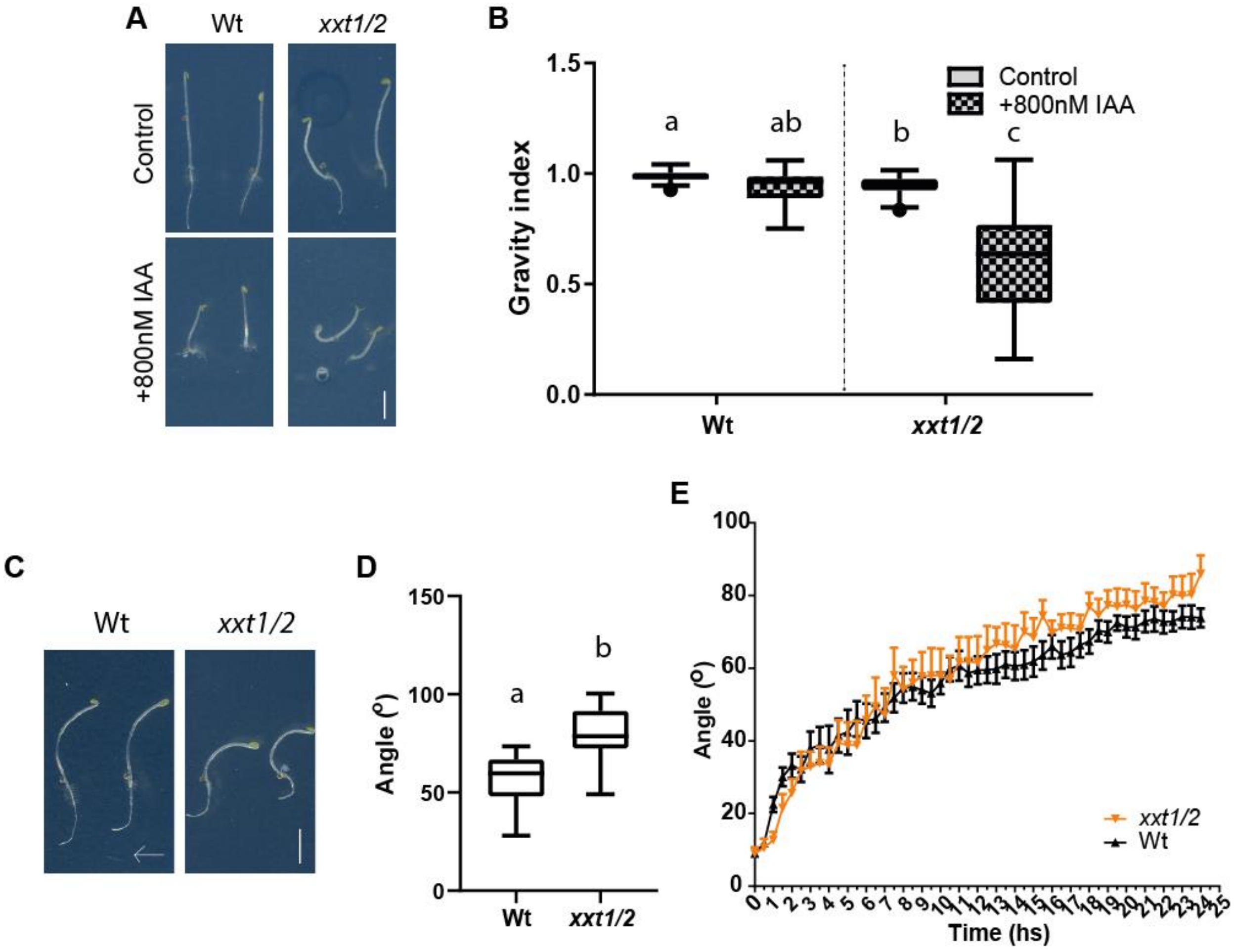


**Figure S10**. Xylosylation of xyloglucans. (**A-B**) Double mutant of xylosyltransferases *xxt1* and *xxt2* were grown on 800 nM indole-3-acetic acid (IAA) or dimethyl sulfoxide [DMSO, (Control)] for five days. (**A**) Representative images of *xxt1*/*2*. Scale bar = 5mm. (**B**) Quantification of the Gravity index of *xxt1*/*2*. Data are mean ± SD. One-way ANOVA followed by Tukey's test with *p*-value <0.05 (n = 3 biological replicates with 25-30 seedlings each). (**C-D**) Response to gravistimulation of *xxt1*/*2*. Five-day-old dark-grown hypocotyls were challenged with a 90° angle change in growth orientation and the end point angle between the apex of the hypocotyls and gravity vector was measured 24 h later. (**C**) Representative images. The arrow marks the gravity vector. (**D**) Quantification of the end point angle. Tukey box-plot (n = 3 biological replicates with 25 – 27 angle measurements). One-way ANOVA followed by Tukey's test with *p*-value <0.05. (**E**) Growth kinetics of *xxt1*/*2*. Five-day old dark-grown hypocotyls were challenged with a 90° angle change in growth orientation and placed in an infrared-based dark-imaging box where their growth was recorded. The angle reached every 30 min was quantified. Non-linear fit to a one-phase association curve. K values for each curve were compared. *p*-value<0.05. Data are mean ± SEM (n = 3 biological replicates with 8–10 seedlings each). Similar letters in the graphs mark no significant statistical difference. Different letters in the graphs mark significant statistical difference with a *p*-value < 0.05.

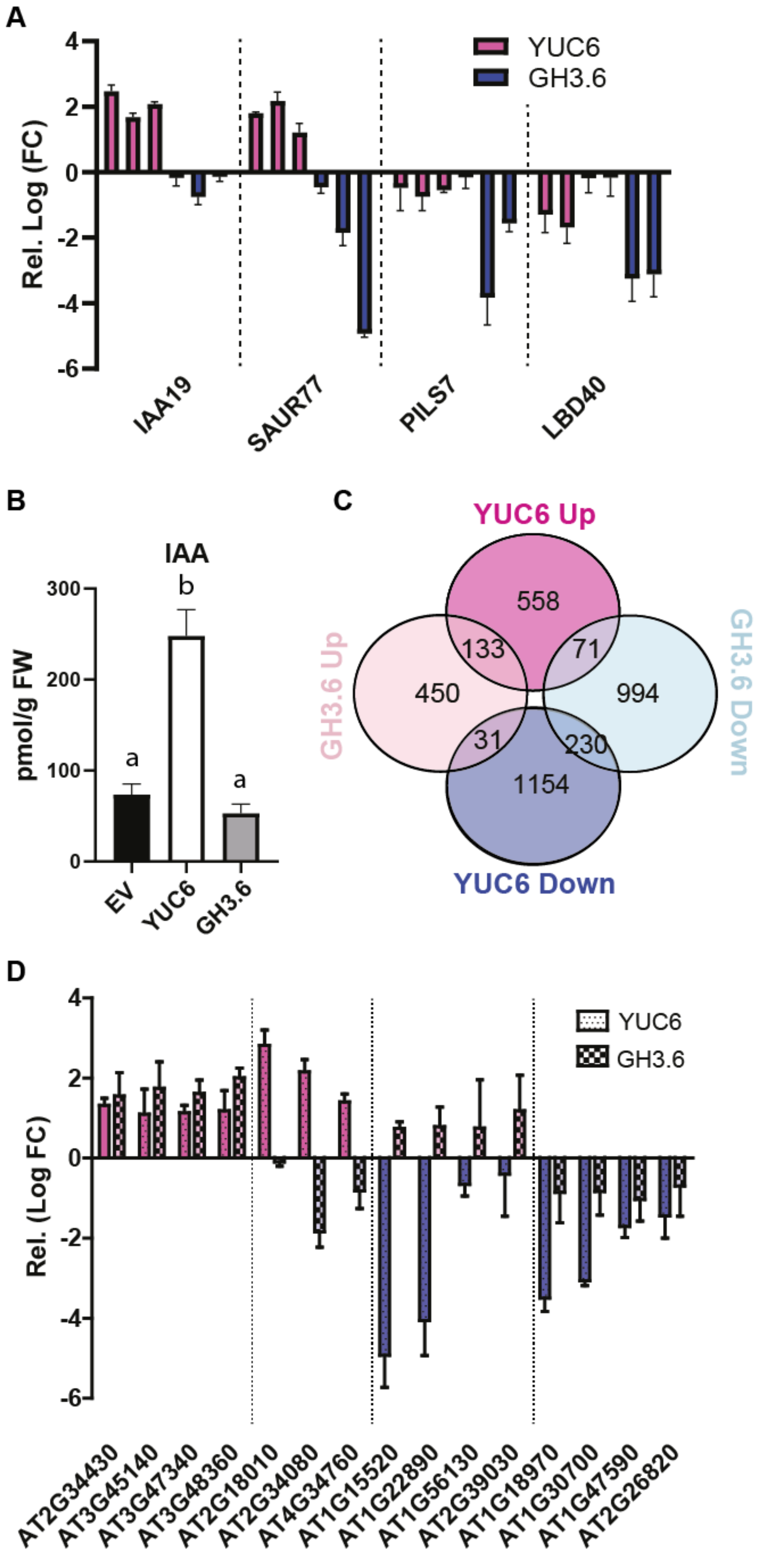
A
YUC6
GH3.6
Rel. Log (FC)
4
2
0
-2
-4
-6
IAA19
SAUR77
PILS7
LBD40
B
IAA
pmol/g FW
300
200
100
0
a
b
a
EV
YUC6
GH3.6
C
YUC6 Up
GH3.6 Up
GH3.6 Down
YUC6 Down
558
133
71
450
994
31
230
1154
D
YUC6
GH3.6
Rel. (Log FC)
4
2
0
-2
-4
-6
AT2G34430
AT3G45140
AT3G47340
AT3G48360
AT2G18010
AT2G34080
AT4G34760
AT1G15520
AT1G22890
AT1G56130
AT2G39030
AT1G18970
AT1G30700
AT1G47590
AT2G26820

**Figure S11.** RNAseq of YUCCA6 (YUC6) and GRETCHEN-HAGEN3.6 (GH3.6) estradiol-inducible lines. (**A**) RT-qPCR of known IAA responsive genes *AUXIN/INDOLE-3-ACETIC ACID19 (IAA19), SMALL AUXIN UP RNA77 (SAUR77), PIN-LIKE7 (PILS7), LOB DOMAIN-CONTAINING PROTEIN40 (LBD40)* after three hours of YUC6 or GH3.6 induction. The $Log_2$ FC values are relative to the treated empty vector control. Each bar represents an independent biological replicate with three technical replicates each. Data are mean ± SD. (**B**) Quantification of free indole-3-acetic acid (IAA) levels of YUC6, G3H.6 and empty vector control (EV) (n = 4 biological replicates). Similar letters in the graph mark no significant statistical difference. Different letters in the graph mark significant statistical difference with a $p$-value < 0.05. (**C**) Venn diagram depicting the number of genes that were differentially up-regulated or down-regulated when compared to the estradiol-treated empty vector control in RNA sequencing of estradiol-inducible lines GH3.6 and YUC6. Three-day old dark-grown hypocotyls were induced for three hours with 10μM β-estradiol. (**D**) Validation by RT-qPCR of a subset of the differentially expressed genes (DEG) belonging to the four categories in (**C**). The $Log_2$ fold change (FC) values are relative to the treated empty vector control. Representative graph. Data are mean ± SD (n = 3 and three technical repeats for each).

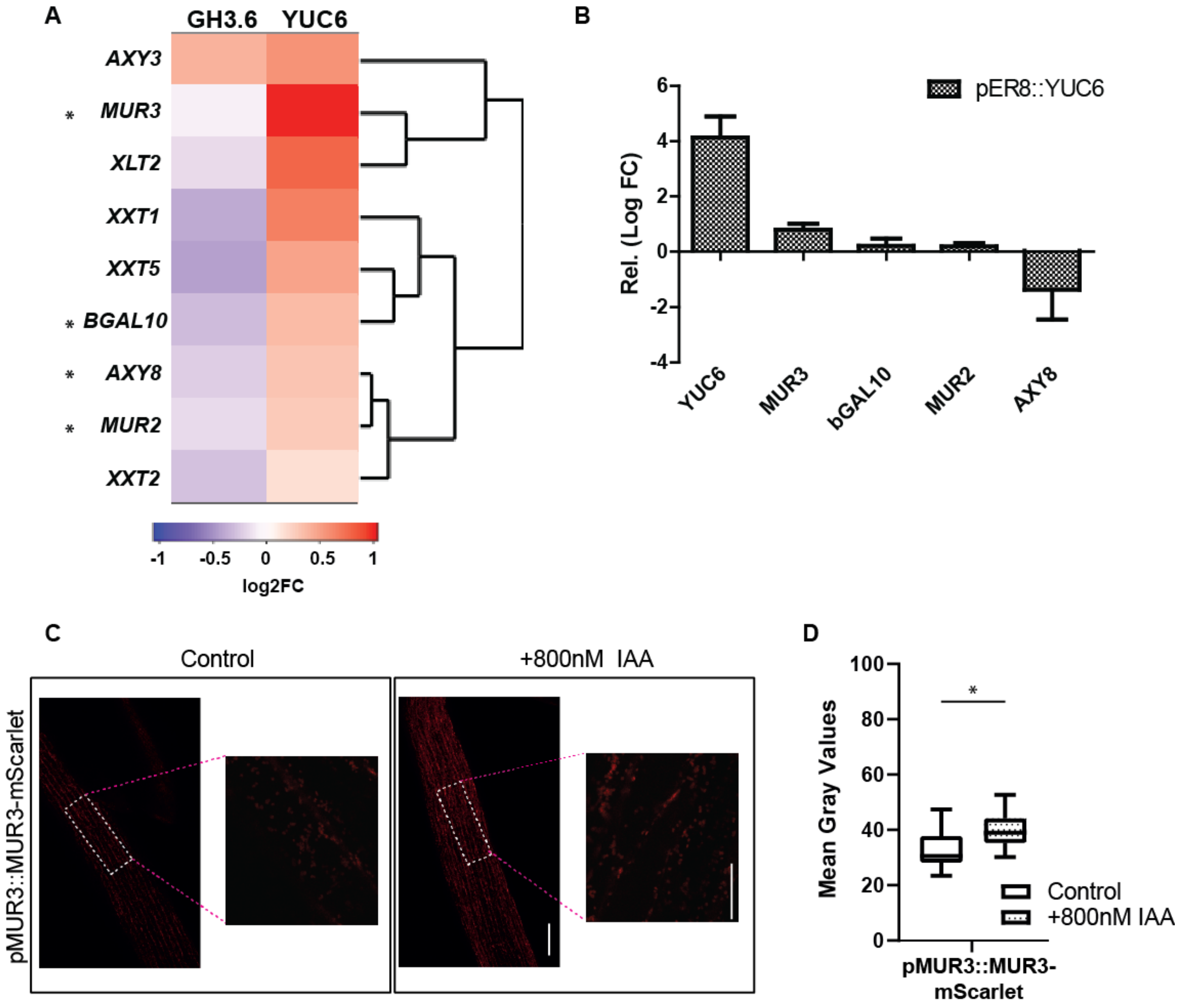


**Figure S12.** Effect of auxin on the transcriptional regulation and protein expression of MURUS3 (MUR3). (**A**) Hierarchical cluster analysis of gene expression of xyloglucan (XyG) biosynthesis genes *ALTERED XYLOGLUCAN3 (AXY3), XYLOGLUCAN L-SIDE CHAIN GALACTOSYLTRANSFERASE POSITION 2 (XLT2), XYLOSYLTRANSFERASE1 (XXT1), XXT2, XXT5, BETAGALACTOSIDASE10 (BGAL10), ALTERED XYLOGLUCAN8 (AXY8)*,and *MURUS2 (MUR2)* under conditions of high (YUC6) and low (GH3.6) endogenous auxin. Analysis of gene expression of XyG biosynthesis genes based on log2 fold change (FC) ratio relative to the empty vector control. Blue represents lowest expression and red highest. Asterisks mark the genes studied in more depth within this work. (**B**) RT-qPCR of XyG genes *MUR3*, *bGAL10*, *MUR2* and *AXY8* in the estradiol-inducible YUC6. Three-day old dark-grown hypocotyls were induced for three hours with 10 µM β-Estradiol. Induction levels of the YUC6 gene were evaluated. The Log2 FC values are relative to the treated empty vector control. Data are mean ± SD (n = 3 and with three technical repeats for each). (**C-D**) Analysis of the changes in protein expression levels of pMUR3::MUR3-mScarlet. Five-day-old dark-grown hypocotyls were treated for 6hs with 800nM indole-3-acetic acid (IAA) or DMSO (Control). (**C**) Representative images of Z-Stacks. Scale bar = 100 µm. White-dotted square marks the measured region of interest (ROI). Scale bar = 25µm in magnified ROI area. (**D**) Quantification of Mean Gray Values. T-test with *p*-value<0.05 (n = 10-12 seedlings). *: *p*-value≤0.05.

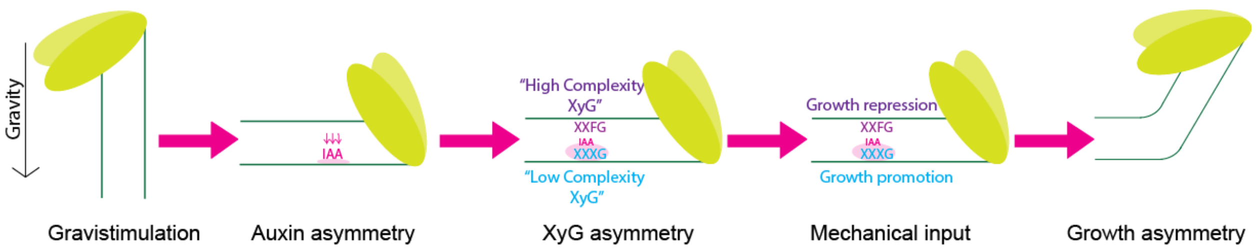


**Figure S13.** Schematic model for the auxin effect on xyloglucan (XyG) composition. After gravistimulation, the first event to take place is the redistribution of auxin to the new direction of the gravity vector (downwards side/convex). This in turn will impact on the composition of the XyG sidechains in a spatially-defined manner. There will be an enrichment in high complexity xyloglucans (XXFG) on the downwards side, while an enrichment of low complexity xyloglucans (XXXG) takes place on the upwards side. Finally, there will be a growth response with cellular expansion and growth promotion taking place on the cells of the downwards side, resulting in the formation of a bend. IAA: indole-3-acetic acid. XyG: Xyloglucan. XyG sidechains 'nomenclature: G (Glucose), X (Glucose+Xylose), L (Glucose+Xylose+Galactose), F (Glu-cose+Xylose+Galactose+Fucose), Ac: Acetyl group.

**Table S1** List of primers used in this study

| Name | Sequence | From/Comment |
|---|---|---|
| RT-qPCR | | |
| ACT2 F | TCGGTGGTTCCATTCTTGCT | [1] |
| ACT2 R | GCTTTTTAAGCCTTTGATCTTGAGAG | [1] |
| AXY8 F | TGATCTTGTGGATCCGTTGA | [2] |
| AXY8 R | GTGCCGGAGCAAATATAGA | [2] |
| bGAL10 | GTCTACGGCGATACTGGTGG | |
| bGAL10 | AGGGACGCTTCTGGGATAGT | |
| GH3.6 F | TCACCACCTATGCTGGGCTTTAC | |
| GH3.6 R | TGAAACCAGCCACGCTTAGGAC | |
| MUR3 F | ATTTCCTTGTGGCGGGTAGG | used for Fig.S12B |
| MUR3 R | CTTGGCAGCCGGTAAGAAGA | used for Fig.S12B |
| MUR3 F2 | CAGCTGGAAGTATGCGTTGC | used for Fig.S9A |
| MUR3 R2 | GAAGAACGGGTCCCACACAT | used for Fig.S9A |
| PILS5 F | TCAGACGGTTACACTTGAAGACA | |
| PILS5 R | GAAATGTAAGTCCCATGTTCACC | |
| YUC6 F | GCTCAGCCTTCTCTCGTTGT | |
| YUC6 R | CGAGGATGAACCGGGAAACA | |
| IAA19 F | GTGGTGACGCTGAGAAGGTT | |
| IAA19 R | CGTGGTCGAAGCTTCCTTAC | |
| SAUR77 F | TCCCACGTCATCGCTCATC | [3] |
| SAUR77 R | AGAACTCAGCCAACTCCTC | [3] |
| PILS7 F | ACCGCCTGCCATGAATATTAGCAC | |
| PILS7 R | AGCACTCATCTTGAGCCACGTC | |
| LBD40 F | GCTGAATCTCAAGCCAACGC | |
| LBD40 R | GACCGGTGTTGAGGAGGTTC | |
| RNAseq Validation | | |
| AT2G34430 F | TTCGCTACCAACTTCGTCCC | |
| AT2G34430 R | GCAACAAACCGGATACACACA | |
| AT3G45140 F | TCTCCAGTTTGATGCCCCAG | |
| AT3G45140 R | CATAAACGGCCGGGTCTAGT | |
| AT3G47340 F | GTTCCGATGATTCTCAGGCCA | |
| AT3G47340 R | TCAGGTCCTCTGTGCCTCAA | |
| AT3G48360 F | GCAAGCGGATGCTTCAACTC | |
| AT3G48360 R | GCAGACACAACCCTTGTCAC | |
| AT2G18010 F | TTTCCGGTTTACGTGGGACC | |
| AT2G18010 R | GAACTCGGAGTGATGGAGCC | |
| AT2G34080 F | CAAGTACCAAGGCCAATGCG | |
| AT2G34080 R | GGCGATCTTAGCCACACCTT | |
| AT4G34760 F | AGAGATGCTCGAGCTTAGGGA | |
| AT4G34760 R | TTGGTACGTCAAGCGGAAGG | |
| AT1G15520 F | TGACGCCTAACCACCACATC | |
| AT1G15520 R | TGGTACTTACGGGACGAGGG | |
| AT1G22890 F | ACAAGTTGGACTAGGCGAGG | |
| AT1G22890 R | AAATTGGAGGGGCCATGGAA | |
| AT1G56130 F | TTGACCGTTGTGATTGATTGTG | |

| | |
|---|---|
| AT1G56130 R | GATCTTCCAAGCCGCGAAAA |
| AT2G39030 F | GAGTCTGGTCTTGCCTCCAC |
| AT2G39030R | CCCCCTTTCTTGAGACGCAT |
| AT1G18970 F | GCAAATCAGCCGTTTCTGCT |
| AT1G18970 R | TCACTTGGCCAACGTTCCAT |
| AT1G30700 F | CCTCAAACTCCGACCCCAAA |
| AT1G30700 R | ACGGAGGAGTAGGAGCCATT |
| AT1G47590 F | CTACAGGTGTCGCGGAGAAG |
| AT1G47590 R | TAGGGCTTGTGGGTCTTGTG |
| AT2G26820 F | ACCAGGCAAAAGGCGTTACC |
| AT2G26820 R | TTACTTATGTCGTCTCCGGGC |
| AT1G29670 F | TCATCAGTCGCTACAGCACC |
| AT1G29670 R | TTGCTGAGTTGATGCGGTCT |

II. Genotyping

| **Name** | **Sequence** | **From** |
|---|---|---|
| *bgal10-1* F | TGTGGTGTTGATGTGGTCAAC | [4] |
| *bgal10-1* R | GCAAGTGAATCTGACTTTCGC | [4] |
| *axy8-1* F | GGGACTCAGGTGGCTAACTT | |
| *axy8-1* R | ACCACTTGCTCCGTAGTTCA | |
| *mur3-3* F | TGCAAACGAAATTAAACATAGGC | [5] |
| *mur3-3* R | GAAGAAGAAACTGATTGGGGC | [5] |

III. Cloning

| **Name** | **Sequence** |
|---|---|
| GH3.6 F for cloning | GGGGACAAGTTTGTACAAAAAAGCAGGCTCGATGCCTGAGGCACCAAAGAT |
| GH3.6 R for cloning | GGGGACCACTTTGTACAAGAAAGCTGGGTCTTAGTTACTCCCCCATTGCT |
| MUR3 promoter F | ATATGGTCTCAACCTACAAACTTCCCCACTCCCAC |
| MUR3 promoter R | ATATGGTCTCATGTTAGAGTGTACGAAGATCACAACAT |
| *pro*MUR3 for BsaI site F | cgagatttggCctcaacaaaaatattaacctatttc |
| *pro*MUR3 for BsaI site R | TTTTGTTGAGGCCAAATCTCGTGCATGATGTCCAAC |
| MUR3 gene F | ATATGGTCTCAGGCTGTATGTTTCCAAGGGTTTCT |
| MUR3 gene R | ATATGGTCTCACTGACTGTGTCTTATCTCTCTG |


1. Zhang, T.; Maruhnich, S.A.; Folta, K.M. Green light induces shade avoidance symptoms. *Plant physiology* **2011**, *157*, 1528-1536.
2. Günl, M.; Neumetzler, L.; Kraemer, F.; de Souza, A.; Schultink, A.; Pena, M.; York, W.S.; Pauly, M. AXY8 encodes an $\alpha$-fucosidase, underscoring the importance of apoplastic metabolism on the fine structure of Arabidopsis cell wall polysaccharides. *The Plant Cell* **2011**, *23*, 4025-4040.
3. Li, Z.-G.; Chen, H.-W.; Li, Q.-T.; Tao, J.-J.; Bian, X.-H.; Ma, B.; Zhang, W.-K.; Chen, S.-Y.; Zhang, J.-S. Three SAUR proteins SAUR76, SAUR77 and SAUR78 promote plant growth in Arabidopsis. *Scientific reports* **2015**, *5*, 12477.

4. Sampedro, J.; Gianzo, C.; Iglesias, N.; Guitián, E.; Revilla, G.; Zarra, I. AtBGAL10 is the main xyloglucan β-galactosidase in Arabidopsis, and its absence results in unusual xyloglucan subunits and growth defects. *Plant physiology* **2012**, *158*, 1146-1157.
5. Kong, Y.; Peña, M.J.; Renna, L.; Avci, U.; Pattathil, S.; Tuomivaara, S.T.; Li, X.; Reiter, W.-D.; Brandizzi, F.; Hahn, M.G.; et al. Galactose-Depleted Xyloglucan Is Dysfunctional and Leads to Dwarfism in Arabidopsis. *Plant Physiology* **2015**, *167*, 1296-1306, doi:10.1104/pp.114.255943.
